# NMR Solution Structure of the N‐Terminal GSPII Domain from the *Thermus Thermophilus* Traffic ATPase PilF and Reconstruction of its c‐di‐GMP Binding Capability

**DOI:** 10.1002/cbic.202400959

**Published:** 2025-03-12

**Authors:** Konstantin Neißner, Carolin Frohnapfel, Heiko Keller, Elke Duchardt‐Ferner, Vanessa Schneider, Zeinab Kamjou, Beate Averhoff, Jens Wöhnert

**Affiliations:** ^1^ Institute for Molecular Biosciences Goethe-University Frankfurt/M. Max-von-Laue-Str. 9, 60438 Frankfurt Germany; ^2^ Center for Biomolecular Magnetic Resonance (BMRZ) Goethe-University Frankfurt/M. Max-von-Laue-Str. 9 60438 Frankfurt Germany; ^3^ Institute for Molecular Biosciences Goethe-University Frankfurt/M Max-von-Laue-Str. 9 60438 Frankfurt Germany; ^4^ Bruker Biospin GmbH &Co. KG Rudolf-Plank-Str. 23 76275 Ettlingen Germany; ^5^ Molecular Microbiology and Bioenergetics Goethe-University Frankfurt/M. Max-von-Laue-Str. 9 60438 Frankfurt Germany

**Keywords:** Protein structure, NMR-spectroscopy, cyclic-di-GMP, *Thermus thermophilus*, Natural transformation

## Abstract

The cyclic dinucleotide c‐di‐GMP is an important second messenger molecule in bacteria and interacts with a variety of receptor molecules including RNA and protein domains. An important class of c‐di‐GMP‐binding protein domains are the general secretory pathway type II (GSPII) domains as exemplified by the N‐terminal domain of the ATPase MshE from *Vibrio cholerae* (MshEN). MshEN binds monomeric c‐di‐GMP via two consecutive copies of a 24‐residue sequence motif, which form a compact 4‐α‐helical bundle. The ATPase PilF from *Thermus thermophilus* regulates pilus formation, motility and DNA‐uptake. Its N‐terminal section contains three consecutive GSPII domains (GSPII‐A‐GSPII‐C) all with considerable sequence homology to MshEN. While the GSPII‐B and the GSPII‐C domains bind c‐di‐GMP, the GSPII‐A domain does not. To determine why it is incapable of c‐di‐GMP‐binding we determined the NMR‐solution structure of this domain. Our structure shows how small deviations in the consensus motif sequence, a stabilizing N‐terminal helical capping motif and intersubdomain interactions absent in MshEN cooperate to prevent c‐di‐GMP‐binding. By combining point mutations and truncations, we re‐established the c‐di‐GMP binding capability. Our findings shed new light on the evolution and functional diversification of GSPII domains and the importance of sequence variations for protein activity in this domain family.

## Graphical abstract

GSPII‐A is the first of three consecutive GSPII domains of PilF from Thermus thermophilus and the only one that does not bind c‐di‐GMP. In this study we solve the structure of GSPII‐A and show the key factors hindering c‐di‐GMP binding. Through mutagenesis, we elucidate the minimal motif conservation needed for c‐di‐GMP recognition.

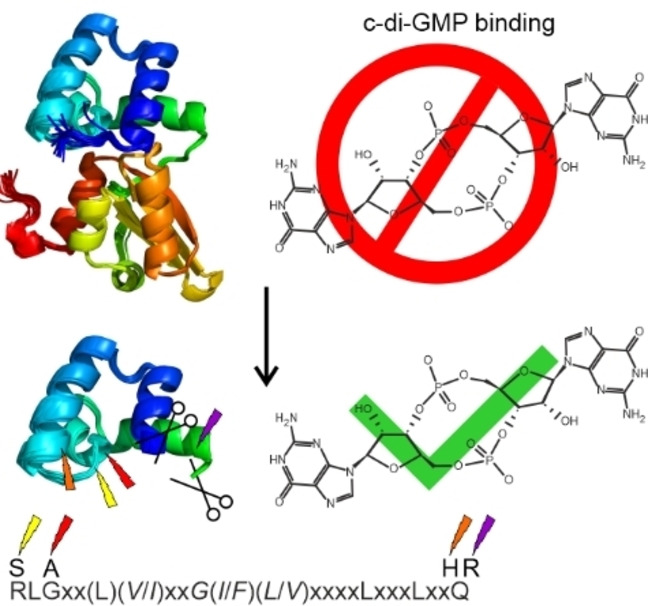

## Introduction

**Figure 1:**
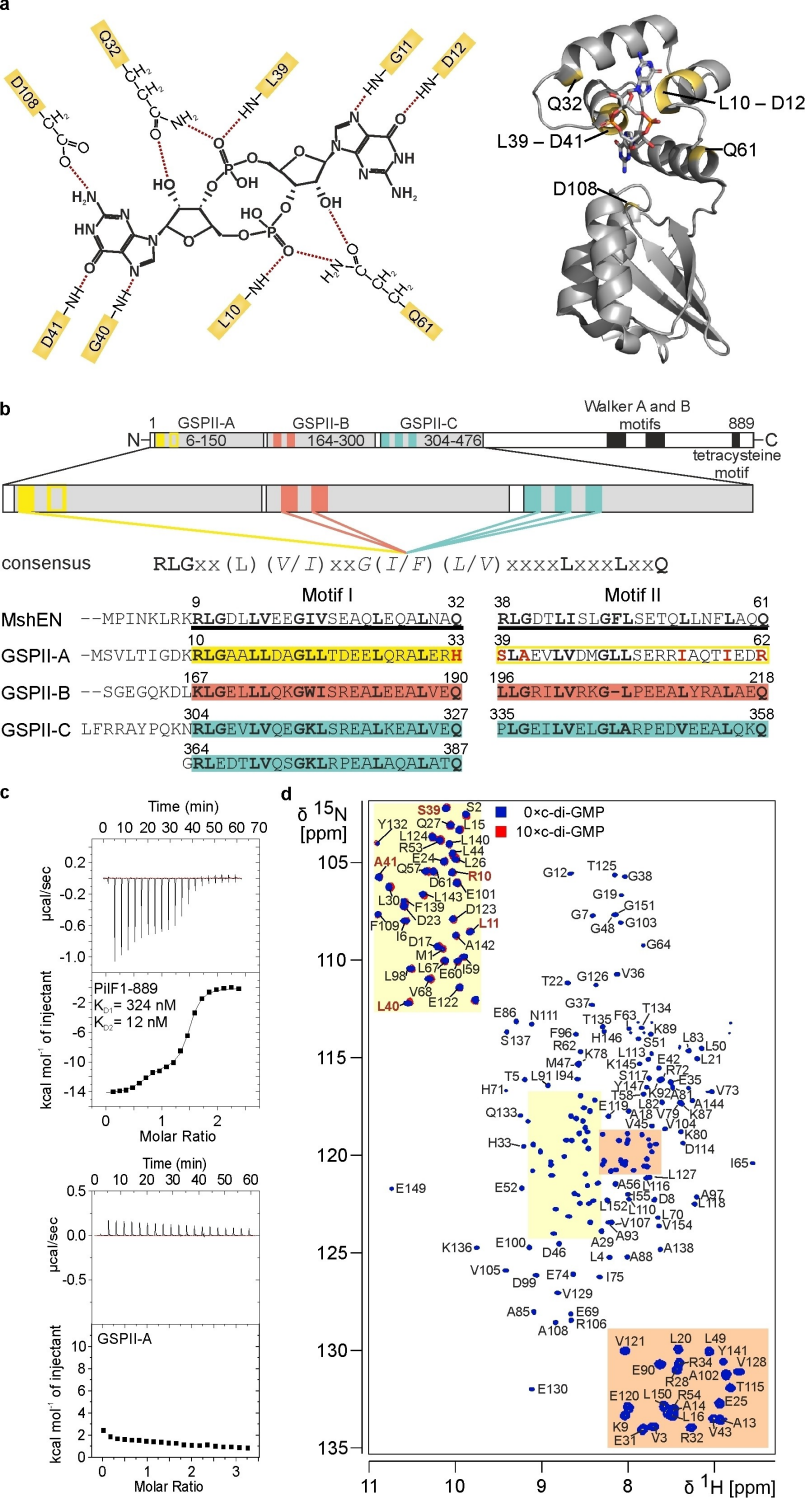
**PilF domain organization, sequence homologies and c–di‐GMP binding**. (**a**) Intermolecular hydrogen bonds for the recognition of c‐di‐GMP in the MshEN‐c‐di‐GMP complex (left). The locations of the amino acids involved in these intermolecular hydrogen bonds are highlighted in the X‐ray structure of the MshEN/c‐di‐GMP complex (right). (**b**) Schematic presentation of the PilF domain organization with the focus on the three N‐terminal GSPII domains and their sequence homologies to each other and to MshEN. PilF_1–154_ (GSPII‐A) shows the lowest sequence conservation with residue differences in key positions highlighted in red. (**c**) ITC‐based c‐di‐GMP binding assays for full‐length PilF (PilF_1–889_) (top) and isolated PilF_1–154_ (GSPII‐A, bottom). (**d**) Backbone amide NMR signal assignments of PilF_1–154_ in a ^1^H, ^15^N‐BEST‐TROSY‐HSQC spectrum at 45 °C at 800 MHz. Resonances of residues at key positions that are directly involved in c‐di‐GMP binding in MshEN are labeled in red in the upper left corner in the absence (blue) and the presence (red) of 10 equivalents of c‐di‐GMP.

For bacteria to thrive in rapidly changing environments quick adaptation to environmental cues such as changing temperatures, fluctuating pH or light conditions as well as alternating salt concentrations is important. To signal these cues bacteria employ a variety of second messenger molecules such as the cyclic nucleotides cAMP and cGMP that are also found in eukaryotes as well as cyclic dinucleotides (CDNs). The most prominent and well‐studied CDN is 3’→5’ cyclic dimeric guanosine monophosphate (c‐di‐GMP). It was originally discovered as an allosteric activator of the bacterial cellulose synthase of *Gluconacetobacter xylinus* ^[1]^ and is now recognized as an ubiquitous second messenger in bacteria.[^2^, ^3^, ^4^] An intricate and widespread network^[5]^ of diguanylate cyclases (DGCs) carrying the conserved GGDEF domain[^6^, ^7^, ^8^] and phosphodiesterases (PDEs) harboring either an EAL[^9^, ^10^, ^11^] or a HD‐GYP^[12]^ domain regulates the cellular concentration of c‐di‐GMP^[17]^ by catalyzing its synthesis[^13^, ^14^] and degradation,^[11]^ respectively. Synthesis and degradation of c‐di‐GMP have been the focus of a number of reviews.[^4^, ^5^, ^15^, ^16^] They are influenced by environmental cues such as for example light,[^17^, ^18^] oxygen[^19^, ^20^] or changes in cell density.[^21^, ^22^] Additionally, a wide variety of cellular responses has been related to changes in c‐di‐GMP concentrations such as initially the regulation of a cellulose synthase and later on the regulation of biofilm formation,^[23]^ bacterial predation,[^24^, ^25^] stress responses,^[26]^ cell‐cycle progression,^[27]^ cell differentiation,^[28]^ motility,^[29]^ the regulation of secretion systems,[^30^, ^31^, ^32^] switching between motile and sessile lifestyles^[33]^ and virulence.^[34]^ To convey all these cellular responses from information comprised in changing cellular c‐di‐GMP concentrations a sophisticated network of c‐di‐GMP receptors and effectors is required to mediate downstream signaling processes. Previous studies have established a molecular basis for downstream c‐di‐GMP signaling by revealing effector proteins and classifying them into motif‐based protein families. However, the large variety of c‐di‐GMP regulated processes suggests that this list is still incomplete.^[35]^ So far these receptors bind intercalated c‐di‐GMP dimers via a degenerate GGDEF type I domain,[^36^, ^37^] monomeric c‐di‐GMP by degenerate EAL domains[^18^, ^38^, ^39^] or monomeric as well as dimeric c‐di‐GMP via PilZ domains[^40^, ^41^, ^42^] and even as intercalated tetramers by motifs with the signature sequence RXD‐X_8_‐RXXD.^[28]^ An intriguing discovery was the c‐di‐GMP mediated ATPase activity inhibition by a direct competition with ATP at the Walker A motif of FleQ.^[43]^ More recently, the group of c‐di‐GMP binding ATPases was expanded by MshE, an AAA^+^ ATPase that binds c‐di‐GMP and contributes to pilus formation in *Vibrio cholerae*.^[32]^ Subsequent structural studies revealed a domain in the N‐terminal half of MshE (MshEN) that binds c‐di‐GMP in its elongated monomeric form with an affinity of ~500 nM via a previously unobserved c‐di‐GMP binding mode^[44.]^ The c‐di‐GMP binding site is located in the N‐terminus of MshEN (Figure 1a) and consists of two consecutive 24 amino acid long sequence motifs (I and II) featuring the highly conserved consensus sequence **RLG**xx(*L*/*V*/*I*)(*L*/*V*/*I*)xx*G*(*L*/*V*/*I*)(*L*/*V*/*I*)xxxx**L**xxxx**L**xx**Q**.^[44]^ The two motifs are separated by a linker of 5 residues. Thus, the complete motif spans ~53 amino acids and is embedded in a tight bundle of four α‐helices (helices α1–α4) representing the N‐terminal subdomain of MshEN and the c‐di‐GMP binding pocket (Figure 1a).^[44]^ Both motifs are engaged in extensive cation‐π stacking (**R**), hydrophobic (**L**) and hydrogen bonding (**G**/**Q**) interactions with the respective GMP moieties (Figure 1a).^[44]^ The backbone amide group of the first leucine residue in each motif is also involved in a hydrogen bonding interaction with the respective phosphate group of c‐di‐GMP in addition to the hydrophobic interactions with the guanine base.^[44]^ The C‐terminal subdomain of MshEN is formed by an arrangement of three α‐helices (helices α5–α7) surrounding a mixed β‐sheet (β‐strands β1–β3). This subdomain is not involved in c‐di‐GMP binding except for a single residue (D108).^[44]^ The overall fold of MshEN identifies this domain as a member of the ‘general secretory pathway II’ or GSPII domain family. However, not all members of this protein domain family bind c‐di‐GMP and/or feature the 24 amino acids consensus sequence motifs.

In previous studies we showed that the traffic ATPase PilF of *Thermus thermophilus* is able to bind c‐di‐GMP linking c‐di‐GMP mediated signaling to natural competence by horizontal gene transfer and cell motility.[^45^, ^46^] PilF is a cytoplasmic, hexameric rotary ATPase of the type II/IV secretion NTPase subfamily^[47]^ that provides energy for the assembly of one of the most efficient natural transformation systems as well as subsequent DNA uptake.[^48^, ^49^, ^50^, ^51^] The multi‐component DNA translocator machinery consists of 16 different proteins and extends from the cytoplasm to the extracellular environment penetrating the inner and outer membrane as well as the peptidoglycan layer in the intermembrane‐space.[^52^, ^53^] Through cryogenic electron microscopy (cryo‐EM) it was shown that hexameric PilF forms a dumbbell‐like complex with a distinct separation of the N‐ and C‐terminal halves that are connected via a stem‐like structure.[^54^, ^55^] However, due to lack of resolution and intrinsic dynamics, a comprehensive structural description of the N‐terminal part of the protein is still missing. The structurally well‐characterized C‐terminal half harbors the ATPase module of PilF that contains a canonical Walker A and an atypical Walker B motif as well as a zinc‐binding tetracysteine motif.[^51^, ^53^] The N‐terminal half of PilF presents the interaction interface with the inner‐membrane platform of the DNA‐translocator by binding to PilM.[^56^, ^57^] It features an unusual arrangement with three successive GSPII domains (Figure 1b) with so far unknown functions.^[50]^ Domain deletion studies revealed an impact on complex stability, transformation efficiency, pilus formation and twitching motility but the functional role of c‐di‐GMP binding remains unclear.^[53]^ Each of the GSPII domains (A–C) are homologous to each other as well as to MshEN and harbor MshEN‐like c‐di‐GMP binding motifs with varying degrees of conservation (Figure 1b). The GSPII‐B (PilF_159–302_) domain exhibits the highest sequence homology to MshEN.[^44^, ^45^, ^46^] We previously showed that the GSPII‐B and ‐C domains each bind c‐di‐GMP and the complete PilF protein is able to bind two c‐di‐GMP molecules.[^45^, ^46^] Thus, the GSPII‐A (PilF_1–154_) domain is apparently unable to bind c‐di‐GMP raising the question why this domain does not bind c‐di‐GMP despite its high sequence homology to its sibling domains. To address this question we solved the NMR structure of isolated PilF_1–154_ (GSPII‐A) in solution. Our structure yielded detailed structural insights into the causes for its inability to bind c‐di‐GMP. Subsequently, we further characterized structural and sequence requirements for c‐di‐GMP binding to the MshEN motif by resurrecting the c‐di‐GMP binding capabilities of the PilF‐GSPII‐A domain in a process of stepwise mutations and modifications.

## Results and Discussion

### Reintroduction of the MshEN Consensus Binding Sequence into PilF_1–154_ does not Restore Binding Affinity towards c‐di‐GMP

Despite the presence of three consecutive GSPII domains (GSPII‐A to GSPII‐C, PilF_6–150_, PilF_164–300_, PilF_304–476_) with high sequence similarity to each other and to MshEN (Figure 1b) one PilF monomer binds only two c‐di‐GMP molecules in separate binding events with distinctly different K_D_ values of 324 nM and 12 nM measured by ITC (Figure 1c). ITC measurements with domain truncation constructs and isolated domains show that these c‐di‐GMP binding events can be assigned to the GSPII‐B and the GSPII‐C domain of PilF, respectively.^[58]^ The isolated GSPII‐A domain construct (PilF_1–154_) did not show any sign of c‐di‐GMP binding during ITC measurements (Figure 1c) suggesting that PilF_1–154_ is either unable to bind c‐di‐GMP or does bind c‐di‐GMP in a low‐affinity regime that cannot be resolved by ITC. We hypothesized that the amino acid deviations in key positions of the consensus sequence from the MshEN‐like c‐di‐GMP binding motif in PilF_1–154_ are the leading cause for its inability to bind c‐di‐GMP. A comparison of the c‐di‐GMP binding motifs of PilF_1–154_ to the GSPII constructs PilF_159–302_ (GSPII‐B), PilF_294–482_ (GSPII‐C) and MshEN, which are all able to bind one c‐di‐GMP molecule with high affinity, reveals that amino acids at key positions in PilF_1–154_ are replaced by residues with different chemical properties in particular in motif II (Figure 1b). In both c‐di‐GMP binding motifs of PilF_1–154_ the last residues – H33 and R62, respectively, differ from the glutamine residues that are present at this position in MshEN, PilF_159–302_ and PilF_294–482_ (Figure 1b). In both MshEN and PilF_159–302_ the side chains of these glutamines are oriented towards the phosphate‐ribose backbone of the bound c‐di‐GMP. This enables the formation of four intermolecular hydrogen bonds and is thus an important contribution to c‐di‐GMP binding (Figure 1a).[^44^, ^58^] While for motif I H33 is the only amino acid deviation from the MshEN consensus sequence, the overall conservation rate is lower for motif II. Besides R62, motif II has four additional residues at key positions that differ from the consensus sequence. S39 at the very start of motif II very likely presents the second most substantial amino acid replacement in regard to the chemical properties compared to the consensus sequence. The arginine residue at the equivalent position in MshEN has been shown to be engaged in a cation‐π stacking interaction with one of the guanine bases of c‐di‐GMP.^[44]^ With serine at this position in PilF_1–154,_ this important binding interaction is potentially absent. The other three differences A41, I55 and I59 present residue changes that retain the hydrophobicity of the corresponding residues in the MshEN consensus sequence and were thus hypothesized to have a smaller impact on binding affinity (Figure 1b).

To understand why PilF_1–154_ does not bind c‐di‐GMP and further elaborate our understanding of the MshEN c‐di‐GMP binding mode, we started by establishing a full NMR signal assignment for all backbone amide groups as well as CO, Cα and Cβ resonance frequencies for this protein. The ^15^N‐BEST‐TROSY‐HSQC spectrum of PilF_1–154_ shows a broad distribution of all expected amide signals for residues 1–154 with minimal signal overlap indicating a well‐folded protein (Figure 1d). Through analysis of the recorded 3D spectra 100 % of the backbone signals (H^N^, N, Cα, Cβ, CO, Hα and Hβ), 99 % of the sidechain aliphatic carbon, 94 % of the aliphatic proton, 71 % of the aromatic carbon and 61 % of the aromatic proton signals were assigned. Adding 10 equivalents of c‐di‐GMP to the PilF_1–154_ sample does not cause any chemical shift changes (Figure 1d) as highlighted for a section of the spectrum containing signals for key residues of the MshEN‐like motifs (Figure 1d, red letters). Thus, PilF_1–154_ does indeed not show any signs of c‐di‐GMP binding in these experiments. In order to analyze if the aforementioned amino acid replacements are the decisive cause for the inability of PilF_1–154_ to bind c‐di‐GMP, we designed a construct that restores the MshEN c‐di‐GMP consensus binding motifs in PilF_1–154_. This MshEN‐like PilF_1–154_ construct ‐ PilF_1–154_ H33Q S39R A41G I55L I59L R62Q – is still a well‐folded protein (Supplementary Figure 1). It was exposed to 15 equivalents of c‐di‐GMP in an NMR‐titration experiment, which revealed no chemical shift changes and consequently no c‐di‐GMP binding (Supplementary Figure 1). Consequently, there must be other structural or dynamical factors at play that interfere with c‐di‐GMP binding in addition to the sequential differences between the c‐di‐GMP binding consensus motifs of PilF_1–154_ and MshEN.

### The NMR Structure of PilF_1–154_ Reveals a Rigid Protein Fold that is Similar to MshEN with Distinct Differences in Subdomain Orientation

In the preceding section, we showed that PilF_1–154_ differs from MshEN in the identity of key residues in the two c‐di‐GMP binding motifs. This alone, however, does not explain the inability of PilF_1–154_ to bind c‐di‐GMP.

**Figure 2:**
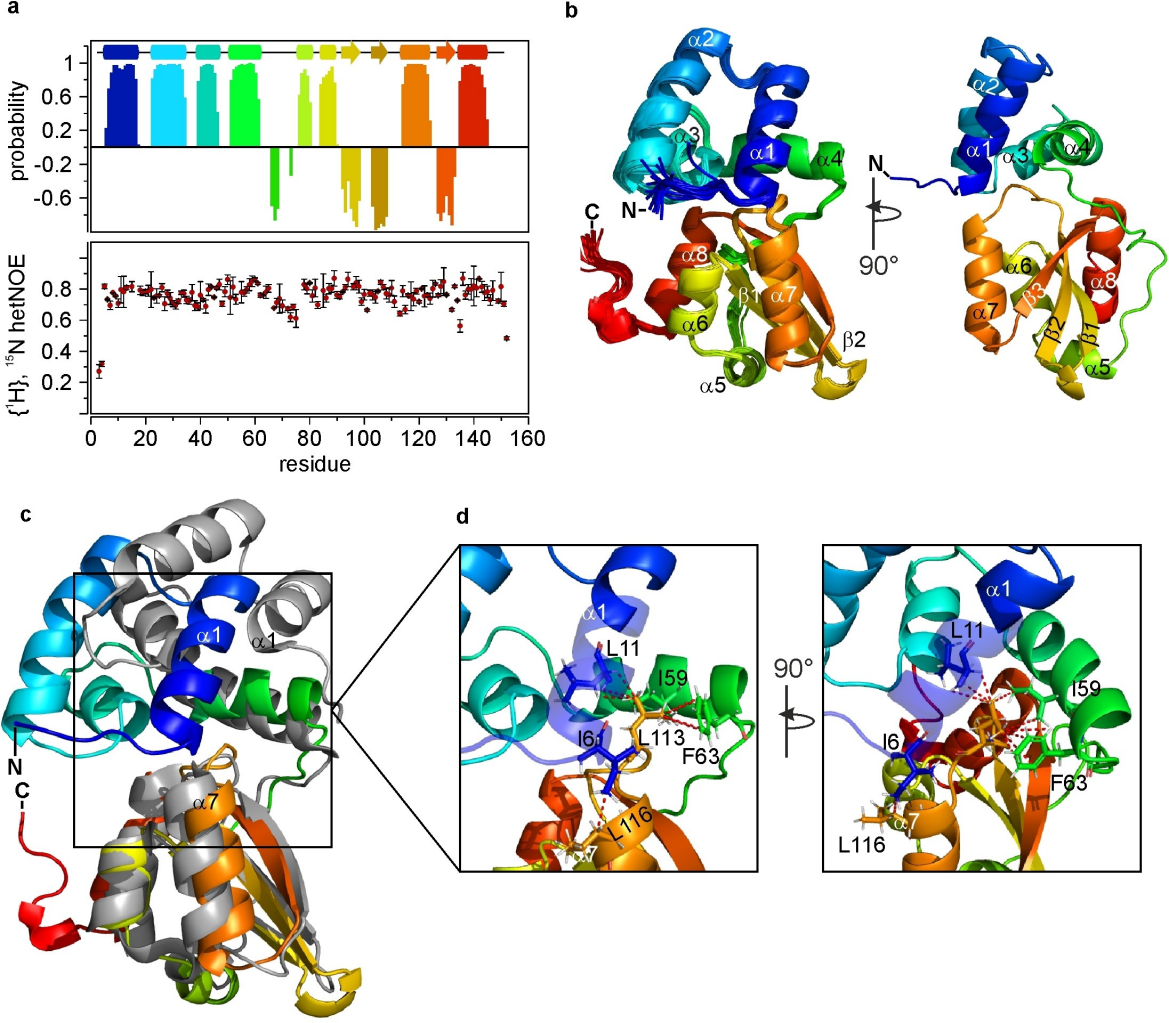
**NMR solution structure of the PilF_1–154_ domain**. (**a**) Chemical shift based secondary structure probability calculated by TALOS‐N (top) and {^1^H},^15^N‐hetNOE data (bottom) plotted against the sequence. The probabilities for α‐helix formation are given as positive numbers, the probabilities for β‐sheet formation are given in negative numbers. The TALOS‐N derived secondary structure elements are shown on top in rainbow colors. (**b**) Solution structure of PilF_1–154_ in cartoon representation. An overlay of the 20 lowest energy structures is shown on the left and the structure with the lowest target function is shown on the right turned 90° to the left. The structures are shown in rainbow colors from blue to red. Secondary structure elements are annotated. (**c**) Alignment of the C‐terminal domains of PilF_1–154_ (rainbow) and MshEN (gray). (**d**) Close‐up of the hydrophobic core formed by residues L11 (helix α1), I59 and F63 (helix α4) of the N‐terminal subdomain with L113 from the C‐terminal subdomain of PilF_1–154_. Important residues are shown in stick representation and NOE‐supported hydrophobic interactions are indicated as red dashed lines.

To understand why PilF_1–154_ is unable to bind c‐di‐GMP we solved its structure using solution NMR. The complete assignment of the backbone resonances was used to derive the secondary structure of the PilF_1–154_ domain. Based on the H^N^, N, CO, Cα and Cβ chemical shifts torsion angles ϕ as well as ψ were calculated and the secondary structure of PilF_1–154_ was elucidated using TALOS‐N^[59]^ and plotted against the amino acid sequence (Figure 2a). High probabilities for the formation of 8 α‐helices and 3 β‐strands in total were calculated which revealed obvious structural similarities to MshEN with regard to the quantity and the position of the secondary structure elements.^[44]^ The first four α‐helices are located in the first 60 N‐terminal residues where the two imperfect c‐di‐GMP binding motifs are located. The C‐terminal residues 77 to 154 form the remaining 4 helices α5–α8 and 3 β‐strands β1–β3 (Figure 2a). They are connected to the N‐terminal part by a 14 amino acid linker. Analyzing the fast scale dynamics of PilF_1–154_ in solution revealed that it acts like a uniformly rigid unit with limited internal flexibility (Figure 2a, Supplementary Figure 2). Only the first 5 N‐terminal and the last 2 C‐terminal residues show {^1^H},^15^N‐hetNOE values lower than 0.6 in agreement with the absence of extended flexible regions in the protein core (Figure 2a). This is supported by ^15^N‐spin relaxation data, which show uniform R_1_ and R_2_ relaxation rates throughout the protein except for the N‐ and C‐termini (Supplementary Figure 2).

A set of NOESY‐experiments optimized for the amide, aliphatic and aromatic moieties, respectively, was recorded with uniformly ^15^N or ^13^C, ^15^N labeled samples in H_2_O and D_2_O. Due to the high abundance of leucine (18 %) and valine (9 %) residues in PilF_1–154_ and the high magnetic sensitivity of methyl group signals, the leucine and valine methyl groups were selectively ^13^C‐labeled and used for additional NOESY‐experiments optimized for the detection of NOEs involving methyl groups. To further increase the structural information from these NOESY‐experiments an additional labeling scheme was used to stereo‐specifically assign the γ^1^ of valine and the δ^1^ of leucine as the pro‐R methyl groups as well as the γ^2^ and δ^2^ as the pro‐S methyl groups as previously described.^[60]^

In sum, this enabled us to calculate a well‐defined structural ensemble representing the solution structure of PilF_1–154_ with an average RMSD of 0.27±0.06 Å and 0.77±0.05 Å for the backbone and all heavy atoms, respectively, for the 20 NMR‐structures with the lowest target functions (Figure 2b). Structures were calculated based on 274 backbone dihedral angle restraints as well as 2369 NOE‐based distance restraints. Detailed NMR statistics are listed in Table 1. Identically to MshEN, the PilF_1–154_ structure can be partitioned into an N‐ and a C‐terminal subdomain (residues 1–65 and 77–154 respectively) with a set of secondary structure elements that is also nearly identical to MshEN.^[44]^ The N‐terminal subdomain is formed by a tightly packed antiparallel arrangement of α‐helices α1–α4. Notably, helix α1 is in close contact with the C‐terminal helix α7 thereby orienting helix α1 and α2 towards the C‐terminal subdomain (Figure 2b). An inflexible linker (residues 64–77) connects the N‐terminal with the C‐terminal subdomain. The C‐terminal subdomain contains a mixed β‐sheet (β1–β3) at its core, which is surrounded by helices α5–α8. The inflexible linker is tightly packed on top of the β‐sheet and also interacts with helix α8. It ends in the two short helices α5 and α6. From there, the two antiparallel β‐strands β1 and β2 continue and are followed by a rather large unstructured loop. This loop ends in helix α7 that tucks in the β‐sheet from the side opposite to the linker. The following β‐strand β3 that is parallel to β‐strand β2 tunnels under the linker and ends in the terminal helix α8 (Figure 2b).

**Table 1:** NMR structural statistics.

Conformational restricting restraints	PilF_1–154_
Total NOE distance restraints	2369
Intraresidual, |*i–j*|=0	619
Sequential, |*i–j*|=1	698
Short range, |*i–j*|<=1	1317
Medium range, 1<|*i–j*|<5	508
Long range, |*i–j*|>=5	544
Dihedral angle restraints (Talos+)	274
No. of restraints per residue	15.4

Residual restraint violations	
Average no. of distance violations per structure	
0.1–0.2 Å	0
0.2–0.5 Å	1
>0.5 Å	0
Average no. of dihedral angle violations per structure	
1–10°	0
>10°	0

Model quality (ordered residues)	
Structures in final ensemble	20
Target function value	2.66±0.12
R.m.s deviation backbone atoms (Å)	0.27±0.06
R.m.s deviation heavy atoms (Å)	0.77±0.05

MolProbity Ramachandran statistics	
Most favoured regions	93.2
Allowed regions	6.8
Disallowed regions	0

Model contents	
Ordered residue ranges (hetNOE >0.6)	5–151
Total No. of residues	154
BMRB accession number	34929
PDB code	9G1W

The overall structure of PilF_1–154_ corresponds to an MshEN‐like fold. However, the RMSD for a global alignment of PilF_1–154_ (residues 6–146) and MshEN of 3.9 Å is rather high (Supplementary Figure 3a).^[44]^ In contrast, an alignment of the individual subdomains from MshEN and PilF_1–154_ results in a good agreement between the subdomains of the respective proteins with backbone RMSD values of 1.7 Å for the N‐terminal and 1.3 Å for the C‐terminal subdomains. The N‐terminal subdomain in MshEN harbors the complete c‐di‐GMP binding motif, which at its core forms two hydrophobic, triangular clusters consisting of the leucine residues L25, L29, L39 as well as L10, L54 and L58 onto which c‐di‐GMP stacks.^[44]^ This characteristic feature can also be found in PilF_1–154_ where leucine residues L26, L30, L40 as well as L11 and isoleucine residues I55 and I59 are arranged into two such triangular hydrophobic clusters with only the sidechain of L11 in a different orientation compared to the equivalent L10 in MshEN (Supplementary Figure 3b). In summary, this demonstrates that the deviations from the c‐di‐GMP‐binding consensus sequence in PilF_1–154_ have no significant structural consequences and highlights the pronounced structural similarities between the individual subdomains of MshEN and PilF_1–154_ down to the level of very specific sidechain placements.

While the individual subdomains of MshEN and PilF_1–154_ are structurally very similar, their relative orientation is distinctly different (Figure 2c). This difference in subdomain orientation is the result of extensive inter‐subdomain hydrophobic interactions in PilF_1–154_ which involve the N‐terminal residues I6 and L11 of helix α1 and I59 and F63 of helix α4 as well as the C‐terminal residues L113 of the irregularly structured loop connecting β‐strand β2 with helix α7 and L116 of helix α7 (Figure 2c, d). L113 is positioned at the core of the subdomain interface forming a hydrophobic cluster with L11, I59 and F63 (Figure 2d). Thus, in combination with the interaction between I6 of helix α1 and L116 of helix α7 this leads to a tilting of the N‐terminal subdomain towards the C‐terminal subdomain. Both of these interactions are directly supported by a well‐defined NOE‐network between proton signals of the involved amino acid residues (Supplementary Figures 4, 5). Interestingly, I6 and L113 are replaced in MshEN by the flexible and positively charged K5 (Figure 1b) and the smaller A110 explaining the absence of the above‐mentioned hydrophobic interaction network and the resulting difference in relative subdomain orientations. Notably, while AlphaFold 3 is rather successful in predicting the structure of the individual subdomains of PilF_1–154_ it fails at predicting the presence of the hydrophobic interactions between the subdomains and in particular the interaction between the F63 and L113 side chains (Supplementary Figure 6) which are directly obvious from the NOE‐data (Supplementary Figure 5).

### An Elongated Helix α1 Stabilized by an Ncap Motif Reaches into the Putative c‐di‐GMP Binding Site

**Figure 3:**
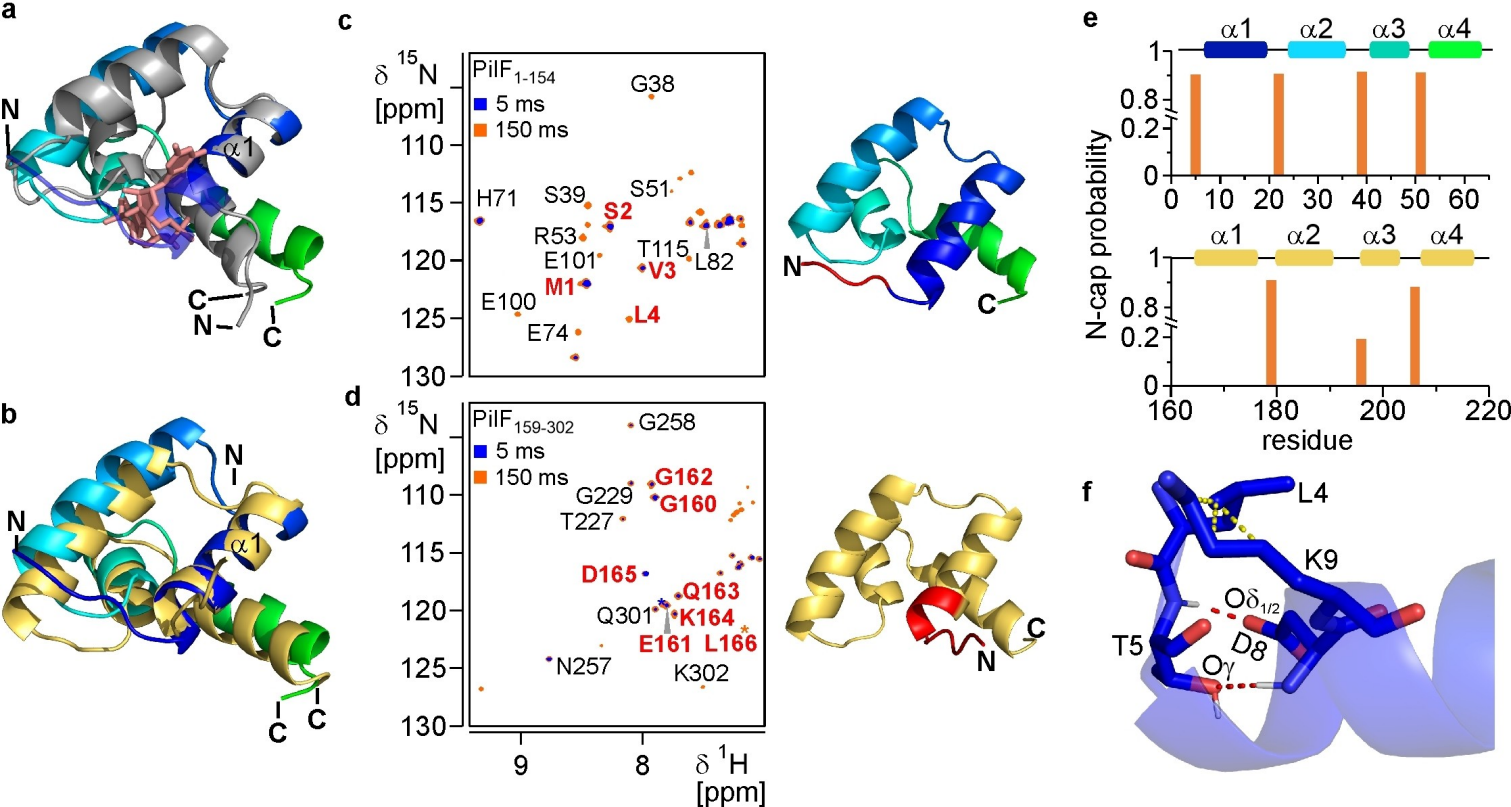
**An elongated and N‐terminally stabilized helix α1 of PilF_1–154_ reaches into the putative c‐di‐GMP binding site**. (**a**) Overlay of the N‐terminal subdomains in PilF_1–154_ (rainbow) and MshEN (gray) with c‐di‐GMP (salmon colored sticks) bound to MshEN. The structures were aligned on the first helix with a backbone RMSD of 0.4 Å (**b**) Overlay of the N‐terminal subdomains in PilF_1–154_ (rainbow colors) and PilF_159–302_ (yellow) in the apo state. The structures were aligned on helix α1 with a backbone RMSD of 0.5 Å. (**c**) CLEANEX‐PM spectra of PilF_1–154_ (left) with mixing times of 5 ms (blue) and 150 ms (red). The first four N‐terminal residues of PilF_1–154_ are labeled in red in the spectra and plotted on the structure (right). (**d**) CLEANEX‐PM spectra of PilF_159–302_ (left) with mixing times of 5 ms (blue) and 150 ms (red). The N‐terminal residues preceding helix α1 (160–163) and the residues forming the first helical turn (164–166) are highlighted in red in the spectra and plotted on the structure (right). All CLEANEX‐PM spectra were recorded with 1 mM uniformly ^15^N labeled samples at 45 °C at 800 MHz. (**e**) Chemical shift based Ncap probability calculation by MISC for the PilF_1–154_ (top) and the PilF_159–302_ N‐terminal subdomain. The respective helices are depicted as barrels with the same color code as in the structure above. (**f**) Close‐up of the Ncap motif stabilizing the elongated helix α1 of PilF_1–154_. Red and yellow dashed lines indicate hydrogen bonding and hydrophobic interactions, respectively.

Another notable structural difference between PilF_1–154_ and MshEN besides the different relative orientation of the two subdomains is the length of helix α1 (Figure 3a). Compared to MshEN helix α1 of PilF_1–154_ is N‐terminally extended by one turn (residues 6–10) and thereby reaches into the putative c‐di‐GMP binding pocket (Figure 3a). For c‐di‐GMP to bind, the first turn of α‐helix α1 would have to unfold so that c‐di‐GMP could fit into the putative binding pocket and form the intermolecular hydrogen bonds and hydrophobic stacking interactions required for c‐di‐GMP binding.^[44]^ Interestingly, in a previous study on the structure of the c‐di‐GMP binding PilF‐domain PilF_159–302_ we showed that in this case a similarly N‐terminally extended helix α1 (Figure 3b) present in the apo state of the protein unfolds by one turn when binding to c‐di‐GMP.^[58]^ Thus, in order to assess the stability of helix α1 and in particular its first N‐terminal turn (residues 6 to 10) for PilF_1–154_ in comparison to the structurally equivalent helix α1 in the apo state of PilF_159–302_ a series of CLEANEX‐PM‐FHSQC experiments with different mixing times were carried out to analyze amide proton‐water exchange rates (Figure 3c–d). The canonical backbone hydrogen bond pattern of α‐helices (NH_i_ to CO_i+4_) protects the majority of helical amide protons from fast exchange with water. However, the first four N‐terminal amide groups of α‐helices are not involved in intrahelical backbone hydrogen bonds and should thus be susceptible to exchange with water. In CLEANEX‐PM‐FHSQC experiments signals are only observed for backbone amide groups that exchange with the solvent water during the mixing time. The fastest exchange with water in this experiment was observed for residues 1–3 in the flexible N‐terminus of PilF_1–154_ preceding helix α1. The signals for their backbone amide groups were already observable at a mixing time of 5 ms in this experiment (Figure 3c, blue). At the longest mixing time technically attainable in this experiment with our equipment – 150 ms – signals for a total of 16 amide groups were observable (Figure 3c, red). Besides the signals for the three N‐terminal amide groups already detected at 5 ms mixing time now signals for L4 and 12 additional surface exposed amide groups throughout the protein are observable. No water exchange on this time scale was detected for residue T5 preceding helix α1 as well as for all other residues of this helix including its first turn (aa 6–10). In contrast, in the PilF_159–302_ domain the first turn of helix α1 unfolds to enable high‐affinity ligand binding. Here, the CLEANEX experiments showed signals of the three residues preceding helix α1 as well as the first three residues from the first turn of this helix already at the short mixing time of 5 ms (Figure 3d, blue). At a mixing time of 150 ms signals for all amide groups in the first turn of this helix are present (Figure 3d, red). These data directly demonstrate that the first turn of helix α1 (residues 6–10) that obstructs the putative c‐di‐GMP binding site is much more stable in PilF_1–154_ than in PilF_159–302_.

Consequently, we took a closer look into the structure of residues 5 to 9 and inspected the chemical environment of the respective amide groups to understand why their protons are protected from exchange. We found that the amide group of I6 is located in the core of a hydrophobic pocket formed by the interaction of I6 and L116 which contributes to the tight interaction between helix α1 and helix α7 (Supplementary Figure 7a). Thus, the backbone amide proton of I6 is protected from exchange. In addition, residues L4 and T5 preceding helix 1 bend over the first helical turn and cover the amides of residues D8 and K9 like a cap (Supplementary Figure 7b).

Furthermore, the web‐based tool MICS, an artificial neural network algorithm that calculates the probability of N‐ and C‐terminal helix capping and β‐turns on the basis of backbone and ^13^Cβ chemical shifts,^[61]^ suggests that based on backbone chemical shifts amino acids 4–10 representing the first turn of helix α1 and its two preceding residues form a classical helix‐stabilizing Ncap motif in PilF_1–154_ (Figure 3e, top, and Supplementary Figure 8a) but not at the equivalent position in PilF_159–302_ (Figure 3e, bottom, and Supplementary Figure 8b). The Ncap motif is a hexapeptide motif with its residues designated as N’‐Ncap‐N1‐N2‐N3‐N4. N1‐N4 are the four N‐terminal residues of an α‐helix, Ncap is the residue immediately adjacent to the helix and N’ the residue preceding the Ncap.^[62]^ In such a motif, the N’ and Ncap residues adopt φ and ψ torsion angles of −102°±12° and 140°±25° and of −86°±12° and 150°±25°, respectively.^[62]^ The Ncap residue is either a threonine, serine or aspartate while the N3 residue is preferably glutamate, glutamine or aspartate. This allows the formation of reciprocal hydrogen bonds between the side chain of the Ncap and the backbone amide of the N3 residue as well as between the backbone amide of the Ncap residue and the side chain of the N3 residue. The structure is further stabilized by hydrophobic interactions between the side chains of N’ and N4. Residues L4(N’)‐T5(Ncap)‐I6(N1)‐G7(N2)‐D8(N3)‐K9(N4) of PilF_1–154_ conform to the sequence requirements for an ideal Ncap motif.[^62^, ^63^] The analysis of our NMR structural ensemble showed that the backbone torsion angles for residues L4 (N’) and T5 (Ncap) also fall into the range reported for Ncap motifs. The side chain hydroxyl group of T5 is hydrogen bonded to the backbone amide group of D8 (N3) while the T5 backbone amide group is hydrogen‐bonded with the D8 side chain carboxylate group as expected for an ideal Ncap structure (Figure 3f). The formation of the latter hydrogen bond is directly supported by the downfield shift of the backbone amide resonance of T5 in comparison with all other threonine backbone resonances (Supplementary Figure 8c). Furthermore, the side chain methyl groups of L4 (N’) pack against the hydrophobic methylene groups of the K9 (N4) side chain (Figure 3f). The presence of all these interactions are supported directly by an extensive network of NOE cross peaks (Supplementary Figure 9).

Thus, in comparison to MshEN the putative c‐di‐GMP binding site of PilF_1–154_ is obstructed by the N‐terminal extension of helix α1 with a very stable helical turn conforming to an ideal Ncap structure. The Ncap structure is further stabilized by hydrophobic interactions with helix α7 in the C‐terminal domain (Supplementary Figure a) that are neither present in MshEN nor in PilF_159–302_. While the apo state of PilF_159–302_ also features an extended helix α1 in this case the extension is unstable since it is not involved in inter‐subdomain interactions and not able to adopt an Ncap structure and can therefore readily unfold to enable ligand binding.

### c‐di‐GMP Binding can be Reestablished in the PilF_1–154_ N‐Terminal Subdomain (PilF_1–65_)

**Figure 4:**
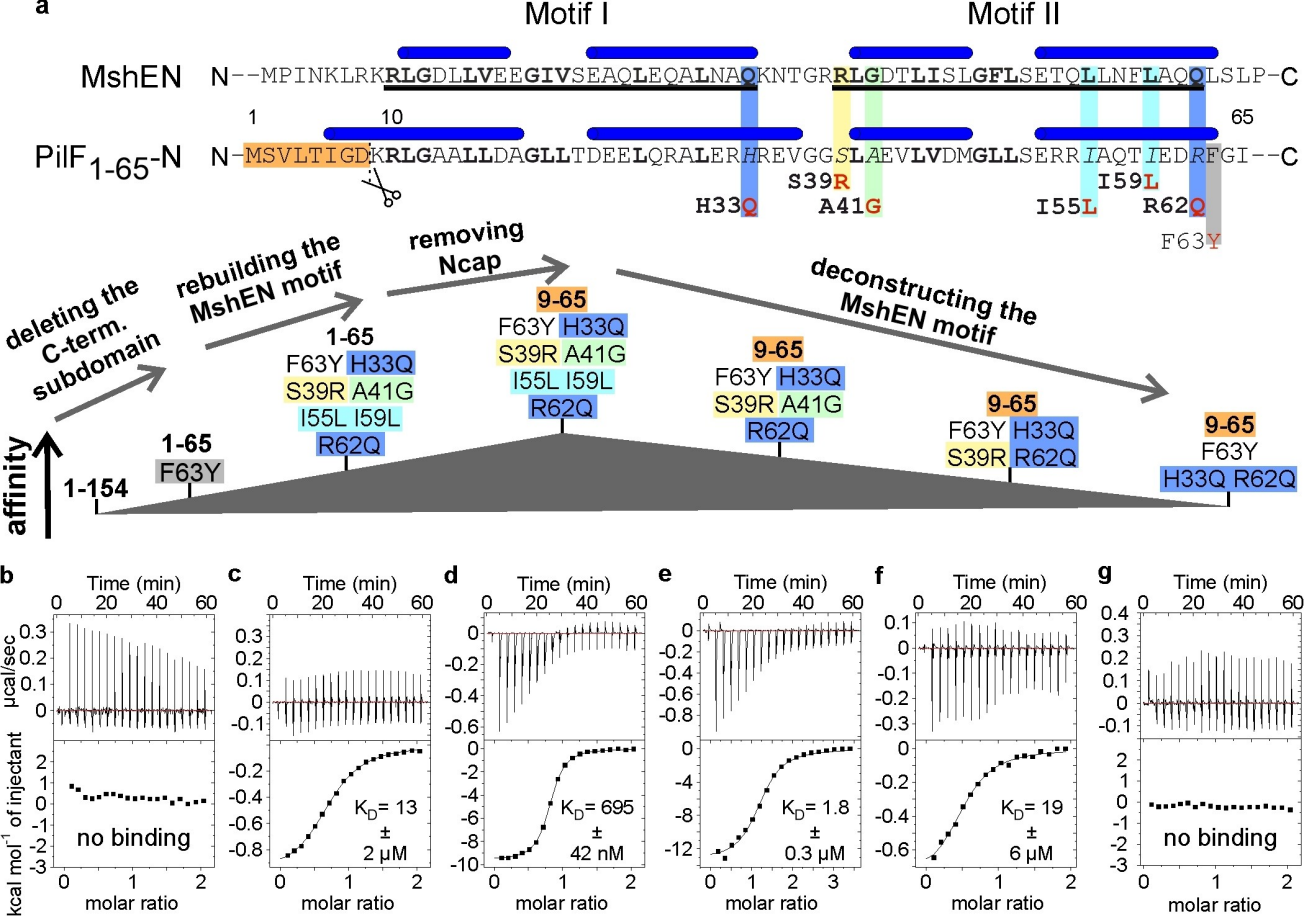
**c‐di‐GMP binding can be re‐established in the isolated N‐terminal subdomain of PilF_1–154_**. (**a**) Sequential alignment of the N‐terminal subdomains of MshEN and PilF_1–154_ with α‐helices displayed as cylinders (top) and a work‐flow scheme (bottom) that shows the different steps and goals in creating the various mutant constructs. Residue differences at key positions are highlighted by colored rectangles and the respective mutations to reintroduce the MshEN sequence are displayed below. The orange rectangle in combination with a cartoon representation of a pair of scissors illustrates the N‐terminal shortening of the PilF_1–154_ N‐terminal subdomain (PilF_1–65_) to create the construct PilF_9–65_. The color code illustrating the mutations is continued in the labeling of the ITC thermograms. (**b**–**g**) ITC thermograms for the binding of c‐di‐GMP to PilF_1–65_ and PilF_9–65_ constructs with varying degrees of completeness of the MshEN consensus sequence for the c‐di‐GMP binding motifs.

In the preceding section, we identified the potential structural underpinnings for the inability of PilF_1–154_ to bind c‐di‐GMP. Based on these findings we created constructs that modify these factors to re‐establish c‐di‐GMP binding. At first, we deleted the C‐terminal subdomain and the intersubdomain linker to remove the tight interactions between the N‐ and C‐terminal subdomains to yield the PilF_1–65_ construct (Figure 4a). Since PilF_1–65_ contains only one aromatic residue (F63) we replaced the solvent exposed F63 with a tyrosine to enable the reliable determination of protein concentrations via UV‐spectroscopy (PilF_1–65_ F63Y). PilF_1–65_ F63Y is a well‐folded protein as indicated by the signal dispersion, the presence of the expected number of amino group signals and the uniform signal intensities in its ^1^H,^15^N‐SOFAST‐HMQC spectrum (Supplementary Figure 10). However, c‐di‐GMP binding is not detectable either in ITC or in NMR titration experiments (Figure 4b and Supplementary Figure 10). In the next round of modifications, we restored a perfect MshEN consensus sequence in PilF_1–65_ F63Y by generating the mutant PilF_1–65_ F63Y H33Q S39R A41G I55 L I59 L R62Q (PilF_1–65_ F63Y mut6). ITC measurements showed that this mutant was now able to bind c‐di‐GMP albeit only with low affinity as indicated by a K_D_ of 13±2 μM (Figure 4c). This was corroborated in NMR‐titration experiments where ligand binding was only observed when the ligand was added in significant excess in agreement with the K_D_ determined in the ITC experiments (see below and Supplementary Figure 11). Remarkably, in the context of the full‐length domain (PilF_1–154_, see above and Supplementary Figure 1) introducing a perfect MshEN consensus sequence was not successful in restoring a c‐di‐GMP‐binding ability to this protein. Thus, this result shows that the interdomain contacts mediated by helix α1 from the N‐terminal and helix α7 from the C‐terminal subdomain are indeed detrimental to c‐di‐GMP binding. However, the K_D_ of PilF_1–65_ F63Y H33Q S39R A41G I55L I59L R62Q (PilF_1–65_ F63Y mut6) for c‐di‐GMP is still ~26fold higher than the K_D_ of MshEN and ~52fold higher than the K_D_ of the isolated N‐terminal subdomain (residue 1–62) of MshEN^[44]^ for the same ligand corresponding to a difference in the free energy of binding of ~1.9 and 2.3 kcal/mol at 20° C, respectively.

While removing the C‐terminal subdomain enables c‐di‐GMP binding, the 26 fold higher K_D_ of PilF_1–65_ F63Y H33Q S39R A41G I55L I59L R62Q (PilF_1–65_ F63Y mut6) for c‐di‐GMP compared to MshEN shows that there are still other factors at work in inhibiting c‐di‐GMP binding. A likely candidate is the presence of the extra turn of helix α1 stabilized by the Ncap motif (Figure 3d). Importantly, in conjunction with a backbone amide resonance assignment of the apo state of PilF_1–65_ F63Y H33Q S39R A41G I55L I59L R62Q (PilF_1–65_ F63Y mut6) CLEANEX experiments showed that the residues in the putative first turn of helix α1 (aa 6–10) are still protected against exchange with the solvent and still have a considerable propensity for α‐helix formation according to their chemical shift index (Supplementary Figure 12). Thus, in the next step the extra α‐helical turn and the Ncap motif were removed in the N‐terminally truncated construct PilF_9–65_ F63Y H33Q S39R A41G I55L I59L R62Q (PilF_9–65_ F63Y mut6). ITC measurements revealed c‐di‐GMP binding to this construct with a K_D_ of 695±42 nM (Figure 4d). This K_D_ is 16 fold lower than the one for PilF_1–65_ F63Y H33Q S39R A41G I55L I59L R62Q (PilF_1–65_ F63Y mut6) which corresponds to a difference in the free energy of binding of 1.6 kcal/mol at 20 °C and is similar to the one of MshEN (K_D_=500 nM).^[44]^ This shows that it is the combination of tight inter‐subdomain interactions with the N‐terminally elongated helix 1 stabilized by an ideal Ncap motif which is responsible for preventing c‐di‐GMP binding to PilF_1–154_ even in the presence of the completely restored consensus sequence. Removing both of these factors in the presence of an ideal consensus motif reestablishes MshEN like c‐di‐GMP binding capabilities as observed in PilF_9–65_ F63Y H33Q S39R A41G I55L I59L R62Q (PilF_9–65_ F63Y mut6) (Figure 4d).

To gain more insight into the impact of individual amino acids from the consensus motif for high‐affinity ligand binding we reverted individual amino acids from PilF_9–65_ F63Y H33Q S39R A41G I55L I59L R62Q back to the WT sequence. The reversion of the I55L and I59L mutants to the WT amino acids I55 and I59 lead to the construct PilF_9–65_ F63Y H33Q S39R A41G R62Q. ITC measurements showed that this construct binds c‐di‐GMP with a K_D_ of 1.8±0.3 μM (Figure 4e) which is only 2.4 fold higher than the one of PilF_9–65_ F63Y H33Q S39R A41G I55L I59L R62Q (Figure 4d) suggesting that the exact identity of these residues is not a decisive factor for the binding affinity as long as large hydrophobic residues are present in this positions.

The additional reversion of the A41G mutation to the WT alanine resulted in the construct PilF_9–65_ F63Y H33Q S39R R62Q. According to ITC experiments this variant binds c‐di‐GMP with a significantly reduced affinity resulting in a K_D_ of 19±6 μM (Figure 4f) which is 30 fold higher compared with the K_D_ for the equivalent construct with the ideal c‐di‐GMP binding motif and 10 fold higher than the K_D_ for the parent construct PilF_9–65_ F63Y H33Q S39R A41G R62Q. The presence of the alanine methyl group instead of the proton found in the glycine residue of the canonical motif might induce a slightly different backbone conformation thereby interfering with hydrogen bond formation between the glycine amide group and the guanine N7 as found in MshEN and PilF_159–302_[^44^, ^58^] In addition, the A41 methyl group is oriented towards the putative c‐di‐GMP binding pocket (Supplementary Figure 13) and could thereby sterically obstruct c‐di‐GMP binding. A complete loss of c‐di‐GMP binding activity is observed in the construct PilF_9–65_ F63Y H33Q R62Q (Figure 4g) where S39 is reintroduced instead of the arginine residue from the canonical binding motif. Thus, out of the six positions in the putative c‐di‐GMP binding motif differing between the PilF_1–154_ sequence and the MshEN derived consensus sequence three positions need to be mutated to restore a measurable c‐di‐GMP binding affinity (S39, H33Q, R62Q) while a fourth mutation is needed to achieve high‐affinity ligand binding (A41G).

### Mutants with a Resurrected c‐di‐GMP Binding Capability Show a Binding Mode Similar to MshEN and PilF_159–302_

**Figure 5:**
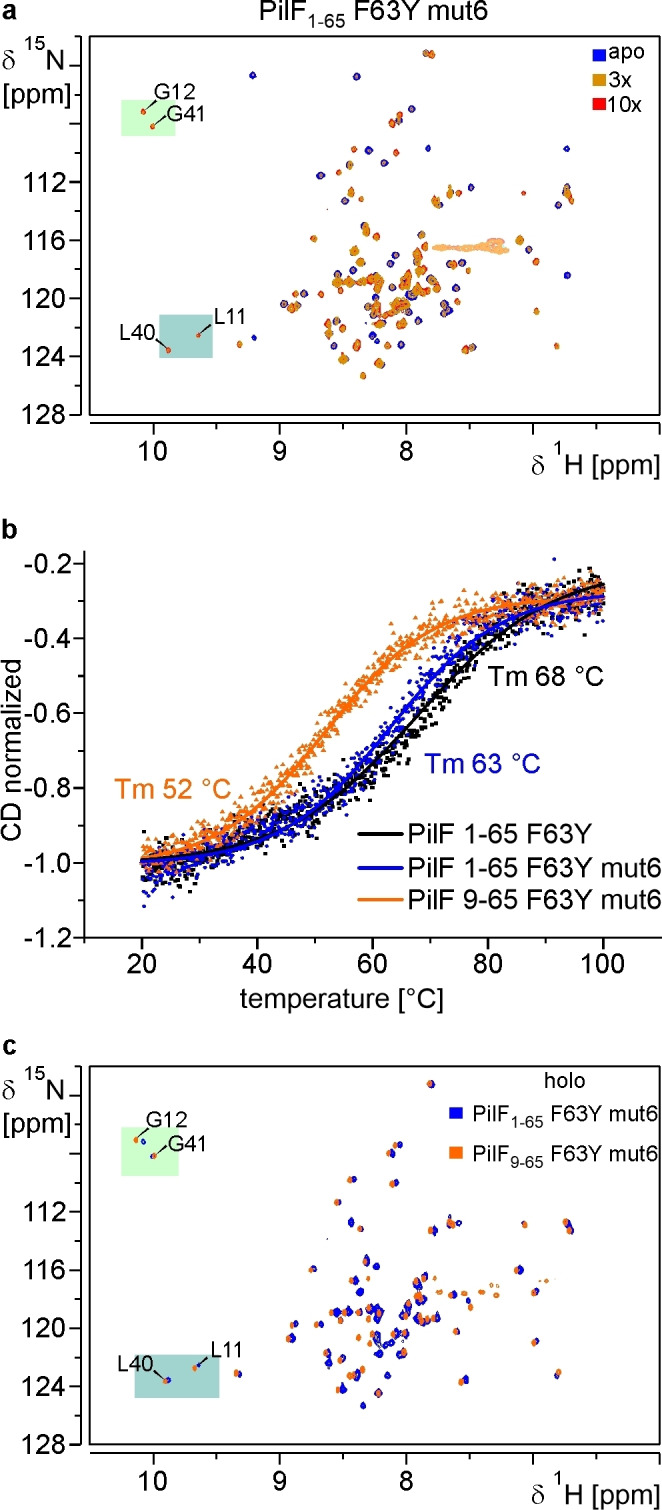
**NMR‐titration experiments yield insights into the c–di‐GMP binding mode of the N‐terminal subdomain constructs of PilF_1–154_**. (**a**) ^1^H,^15^N‐sofast‐HMQC spectra of PilF_1–65_ F63Y H33Q S39R A41G I55L I59L R62Q (PilF_1–65_ F63Y mut6) without c‐di‐GMP (blue) and in the presence of 3 (orange) or 10 equivalents (red) of c‐di‐GMP at 800 MHz. The resonances highlighted by the colored rectangles are corresponding to residues in MshEN that are directly involved in hydrogen bonding interactions with c‐di‐GMP. (**b**) CD‐derived melting profiles for PilF_1–65_ F63Y (black), PilF_1–65_ F63Y mut6 (blue) and PilF_9–65_ F63Y mut6 (orange) with a temperature range from 20 °C to 100 °C. Curve fits to a single unfolding transition are shown and melting temperatures are indicated. (**c**) Overlay of the ^1^H,^15^N‐SOFAST‐HMQC spectra of PilF_1–65_ F63Y mut6 and PilF_9–65_ F63Y mut6 in the presence of saturating amounts of c‐di‐GMP at 950 MHz.

While the affinity measurements for the protein variants described in the previous section showed that we were able to reestablish c‐di‐GMP binding it is initially unclear if the resulting binding mode also corresponds to the one observed in MshEN and PilF_159–302_. Thus, we conducted additional NMR experiments with these constructs to answer this question. The ^1^H,^15^N‐SOFAST‐HMQC spectrum of construct PilF_1–65_ F63Y H33Q S39R A41G I55L I59L R62Q (PilF_1–65_ F63Y mut6) in the apo state shows a broad dispersion of all amide resonances with rather uniform signal intensities and the expected number of signals (Figure 5a). Only upon addition of a high excess of ligand (10fold) large chemical shift changes for many signals were observed clearly indicative of c‐di‐GMP binding (Figure 5a). Strongly downfield shifted signals are observed for the backbone amide groups of L11 and L40 as well as for G12 and G41, respectively. In MshEN and PilF_159–302,_ the leucine residues at the equivalent positions are involved in forming intermolecular hydrogen bonds between their backbone amide groups and the phosphodiester groups of the ligand while the equivalent glycine residues form hydrogen bonds between their backbone amide groups and the N7 nitrogens of the guanine bases (see Figure 1a). For PilF_159–302,_ we recently demonstrated that the extreme downfield proton chemical shifts of these four backbone amide protons are connected with the formation of the intermolecular hydrogen bonds described above.^[58]^ Thus, these intermolecular hydrogen bonds are likely present in the PilF_1–65_ F63Y H33Q S39R A41G I55L I59L R62Q – c‐di‐GMP complex as well.

The ^1^H,^15^N‐SOFAST‐HMQC spectrum of the N‐terminally truncated PilF_9–65_ F63Y H33Q S39R A41G I55L I59L R62Q construct in the apo state differs significantly from the one of the longer PilF_1–65_ F63Y H33Q S39R A41G I55L I59L R62Q (PilF_9–65_ F63Y mut6) construct (Supplementary Figure 14). It shows only 42 of the 56 expected backbone amide group signals. However, these signals are well dispersed across the spectrum. Furthermore, their intensities are much less uniform than the ones for the longer construct. Overall, this is typical for a folded protein with increased dynamics in parts of the structure. It is conceivable that the shortening of the helix α1 in this construct and the deletion of the Ncap motif destabilizes this part of the structure causing the increased dynamics. This is borne out in CD‐melting experiments. There we find that the melting point of PilF_9–65_ F63Y H33Q S39R A41G I55L I59L R62Q is reduced by 11 K compared to the longer PilF_1–65_ F63Y H33Q S39R A41G I55L I59L R62Q and by 15 K compared to PilF_1–65_ F63Y (Figure 5b). On the other hand, the ^1^H,^15^N‐SOFAST‐HMQC spectrum of the PilF_9–65_ F63Y H33Q S39R A41G I55L I59L R62Q – c‐di‐GMP complex is very similar to the one of the equivalent PilF_1–65_ construct (Figure 5c) in particular with regard to the chemical shifts of the backbone amide signals for L11, L40, G12 and G41, respectively. This indicates that also in this complex the corresponding intermolecular hydrogen bonds between the backbone amide groups and the ligand are formed (Figure 1b). The chemical shift index based on a complete backbone NMR resonance assignment for this construct in the c‐di‐GMP‐bound state (Supplementary Figure 15) also demonstrates that in the complex helix α1 is folded.

**Figure 6:**
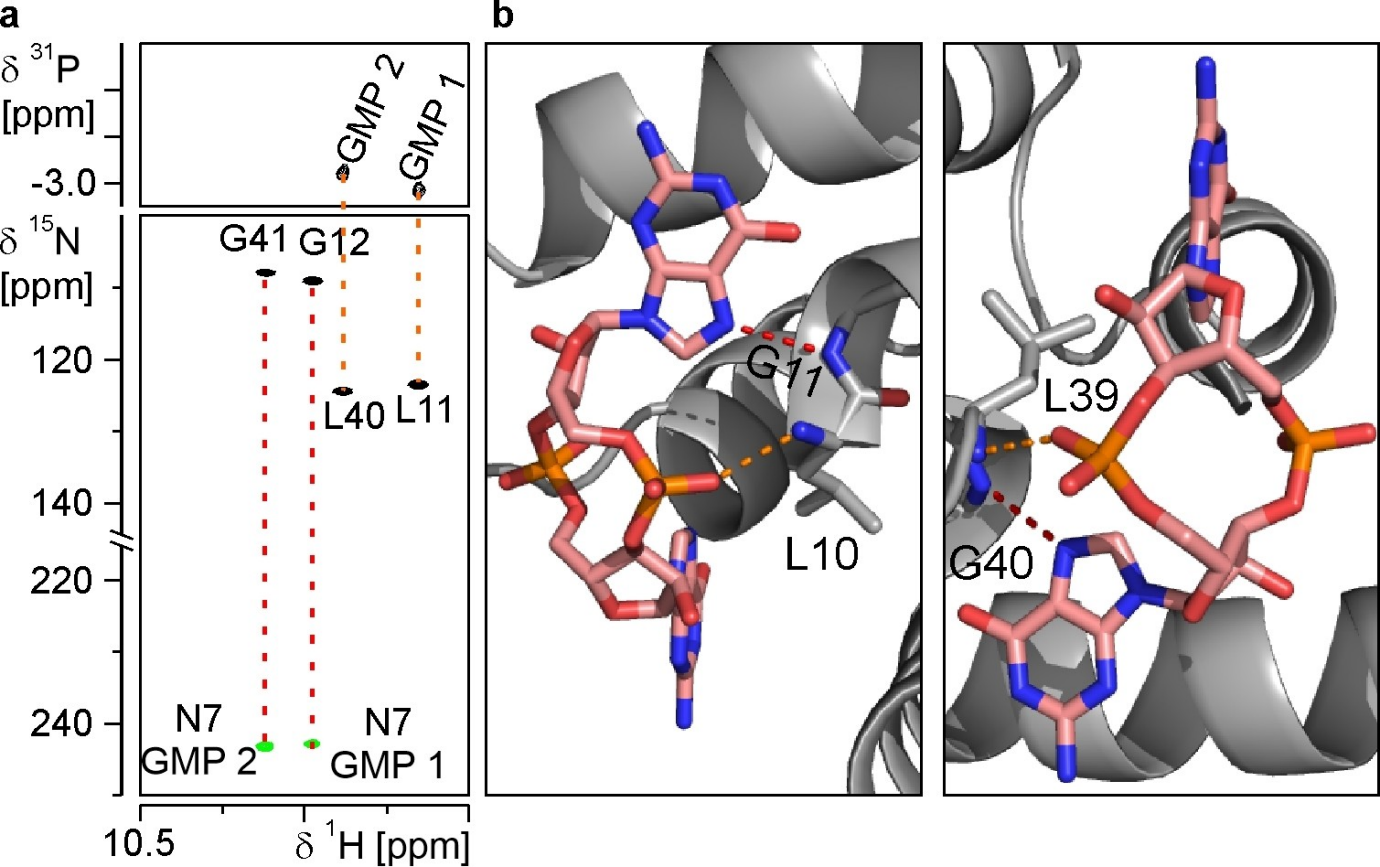
**Directly detected intermolecular hydrogen bonds support an MshEN‐like c‐di‐GMP binding mode**. (**a**) A 2D ^1^H, ^31^P‐SOFAST‐HMQC spectrum at 700 MHz (top) and a 2D BEST‐TROSY‐HNN‐COSY spectrum at 600 MHz for the PilF_9–65_ F63Y H33Q S39R A41G I55L I59L R62Q – c‐di‐GMP complex. Magnetization transfers across the respective hydrogen bonds are indicated as red and orange dashed lines (**b**) Close up of the MshEN c‐di‐GMP binding pocket showing the protein backbone to ligand hydrogen bonds detected in the experiments shown in (**a**). The hydrogen bonds are indicated as orange and red dashed lines, respectively.

The presence of the signature intermolecular hydrogen bonds between the backbone amide groups of the leucine residues L11 and L40 and the two phosphodiester groups of the ligand on the one hand and the backbone amide groups of the glycine residues G12 and G41 and the N7 nitrogens of the ligand on the other hand can also be detected directly by demonstrating the presence of scalar coupling across these hydrogen bonds in dedicated NMR‐experiments.[^64^, ^65^, ^66^] Accordingly, a 2D ^1^H,^31^P‐SOFAST‐HMQC spectrum showed correlations between the backbone amide protons of G12 and G41 and the two ^31^P signals of the ligand (Figure 6). Furthermore, a 2D HNN‐COSY experiment demonstrated the presence of scalar couplings across hydrogen bonds between the backbone amide groups of G12 and G41 and nitrogen resonances with chemical shifts at ~244 ppm which can be assigned to the two N7 nitrogens of the guanine bases of the ligand (Figure 6). Similar correlations were previously observed in experiments with PilF_159–302_ and c‐di‐GMP.^[58]^ Overall, these experiments directly demonstrate that PilF_9–65_ F63Y H33Q S39R A41G I55L I59L R62Q binds c‐di‐GMP with a binding mode closely resembling the one found in MshEN and PilF_159–302_ as indicated by the presence of a very similar intermolecular hydrogen bonding pattern and similar chemical shift characteristics.

NMR experiments with PilF_9–65_ F63Y H33Q S39R A41G R62Q where the leucine residues at positions 55 and 59 of the ideal binding motif are reverted back to the native isoleucines show a behavior of this construct that closely resembles the one of PilF_9–65_ F63Y H33Q S39R A41G I55L I59L R62Q both with regard to the destabilization of the apo state described above as well as with regard to the ligand induced folding and the appearance of the downfield shifted signals for L11, L40, G11 and G42 (Supplementary Figure 16). Thus, the exact identity of these residues is not important for the binding mode in agreement with the very similar K_D_ for the ligand as measured by ITC (see Figure 4) for these two constructs.

In contrast, the additional reversion of the G41 from the ideal binding motif back to alanine in the PilF_9–65_ F63Y H33Q S39R R62Q construct does not only further diminish the affinity of this construct as discussed above (see Figure 4). It also changes the ligand binding mode. While the NMR titration experiments clearly show saturated ligand binding at a high excess of the ligand (Supplementary Figure 17) and ligand‐induced folding, the ^1^H,^15^N‐HMQC spectrum of the complex differs significantly from the equivalent spectra of the previously discussed constructs. This is in line with the suggested sterical interference of the alanine side chain methyl group in the putative ligand pocket.

## Conclusions

Here we elucidated the solution structure of the N‐terminal GSPII‐A domain (PilF_1–154_) of the traffic ATPase from *Thermus thermophilus*. Despite extensive sequence homology to the prototypical c‐di‐GMP binding MshEN domain from *Vibrio cholera* and the other two c‐di‐GMP binding GSPII domains of PilF this domain is unable to bind c‐di‐GMP. Our structure shows that the similarity of these domains on the sequence level is also reflected on the tertiary structure level. However, PilF_1–154_ differs from the previously structurally characterized MshEN and PilF_159–302_ domains in important structural details. This includes an extended N‐terminal helix α1 in the N‐terminal subdomain. This helix reaches into the potential c‐di‐GMP binding site of PilF_1–154_ and is N‐terminally stabilized against unfolding by an ideal Ncap motif. Furthermore, the N‐terminal tip of this helix is involved in intersubdomain interactions with helix α7 from the C‐terminal subdomain. These interactions induce a relative orientation of the two subdomains in PilF_1–154_ that is different from the one found in the c‐di‐GMP binding GSPII domains mentioned above. Amino acids critical for the formation of both the Ncap itself and the intersubdomain interactions in PilF_1–154_ are not conserved in either MshEN or in PilF_159–302_ thereby directly rationalizing these conformational differences.

On the other hand, sequence deviations from the 24 amino acid consensus c‐di‐GMP binding motifs have no direct consequences for the structure of the protein domain but either lead to a direct loss of potential intermolecular interactions with the ligand or contribute to sterically obstructing the potential ligand binding pocket as e. g. in the case of A41. By attempting to resurrect the c‐di‐GMP binding ability of PilF_1–154_ by targeted mutations and modifications, we could show that all three of these structural factors contribute to prevent high‐affinity c‐di‐GMP binding to this domain. Importantly, even restoring the ideal consensus motifs did not lead to high‐affinity c‐di‐GMP binding as long as the intersubdomain interactions and the Ncap motif of helix α1 were left intact. The ability to bind c‐di‐GMP in the prototypical binding mode observed in the MshEN‐c‐di‐GMP complex was only restored when these interactions were also removed. Additional mutation experiments then underscored the importance of individual residues in the consensus motifs for c‐di‐GMP such as the glycine residue at its third position but also showed that there is some additional flexibility in the consensus motif with regard to the identity of amino acids in other positions. Overall, our study helps to clarify the structural determinants for c‐di‐GMP binding in GSPII domains in general and will therefore be helpful in order to predict potential c‐di‐GMP binding activities for these domains in other protein contexts.

Previous deletion experiments have shown that the absence of the PilF_1–154_ domain in PilF *in vivo* causes a decrease in twitching motility for *Thermus thermophilus* cells but does not influence the uptake of environmental DNA nor the ATPase activity of PilF.^[53]^ Interestingly, a similar phenotype was observed when the c‐di‐GMP binding capability of PilF_159–302_ was knocked‐out. On the other hand, the absence or the presence of the PilF_1–154_ domain has no influence on the c‐di‐GMP binding affinity of PilF_159–302_ or full‐length PilF^[58]^ arguing against a role of this domain as an allosteric regulator in PilF function. Together with our structural data this suggests that the PilF_1–154_ domain might act as a structurally stable ‘landing pad’ for recruiting additional proteins important for the function of PilF in powering the twitching motility of *Thermus thermophilus*. Here, the two N‐terminal GSPII domains might form a composite interface for some of their targets. It will be interesting to identify potential binding partners by using PilF_1–154_ or a tandem construct with both N‐terminal GSPII domains as bait in targeted ‘pull‐down’ experiments to gain additional insights into the biological function of this domain and its potential role in twitching motility.

## Experimental Section

### Plasmids and Construct Design

To re‐establish and characterize the MshEN c‐di‐GMP binding motifs in PilF_1–154_ as well as creating shortened constructs a commercially obtained pET‐11a vector containing the PilF_1–154_ coding sequence (GenScript, New Jersey, USA) was used as a starting point for modifications in the coding sequence. The original and all modified pET‐11a vectors code for an N‐terminal hexahistidine tag (His‐6), a TEV‐cleavage site and the respective protein coding sequence in this order.

For the creation of the N‐ and C‐terminally shortened constructs (PilF_1–65_ and PilF_9–65_) standard Gibson assembly was performed with the His‐6‐PilF_1–154_‐pET‐11a vector (GenScript, New Jersey, USA) as the template.^[67]^

Single or multiple amino acid replacements were introduced to the respective vectors (PilF_1–154_, PilF_1–65_ and PilF_9–65_) by site‐directed in vitro mutagenesis with a two‐step protocol as described.^[68]^ All constructs of PilF_1–65_ and PilF_9–65_ contain the F63Y mutation at the very C‐terminus to enable protein detection and concentration determination via UV light absorption measurements at 280 nm due to the lack of aromatic amino acids in the PilF_1–65_ and PilF_9–65_ WT sequences except for F63.

### Protein Expression and Purification

For structure determination and ligand binding assays PilF_1–154_ WT and all mutant constructs were heterologously expressed in chemically competent *Escherichia coli* BL21 [DE3] Gold cells. For PilF_1–154_ WT the commercially obtained His‐6‐PilF1–154‐pET‐11a vector (GenScript, New Jersey, USA) was used. Various constructs differing from the WT sequence of PilF_1–154_ and the terminally truncated versions PilF_1–65_ as well as PilF_9–65_ used in this study were produced by using modified pET‐11a vectors described in the previous section. The expression and purification for unlabeled and uniformly ^15^N or ^13^C,^15^N labeled PilF_1–154_ WT as well as modified PilF_1–154_ constructs with single or multiple amino acid replacements was performed as previously described for PilF_159–302_.^[45]^ Expression of all shortened constructs including amino acid replacements (PilF_1–65_, PilF_9–65_) was identical to PilF_1–154_ WT but no heat‐shock step was included in the purification.

To enhance the quality of the structural data gained by NMR experiments samples with selectively and stereo specifically ^13^C labeled leucine (L) and valine (V) methyl groups (δ1, δ2 and γ1, γ2 respectively) were prepared. Stereo‐specific ^13^C labeling of the L and V methyl groups was achieved by using a mixture of 10 % ^13^C labeled and 90 % unlabeled glucose as the sole carbon source in the ^15^N‐M9‐medium.^[60]^ To selectively ^13^C‐label L and V methyl groups 120 mg/l of the L and V precursor Keto‐3‐(methyl‐^13^C)‐butyric‐4–^13^C acid sodium salt (SigmaAldrich) was added to the ^15^N‐M9‐medium one hour prior to the induction of protein production.^[69]^

Unlabeled and ^13^C,^15^N labeled (3’‐5’) c‐di‐GMP used during this study was enzymatically synthesized with unlabeled or ^13^C,^15^N labeled GTP as a substrate by using a constitutively active mutant of a diguanylate cyclase originating from the thermophilic bacterium *Thermotoga maritima*.[^70^, ^71^]

### Isothermal Titration Calorimetry

The c‐di‐GMP binding capabilities of the various PilF_1–154_, PilF_1–65_ and PilF_9–65_ constructs were characterized by isothermal titration calorimetry (ITC) measurements in three independent titrations for each construct with detectable binding to c‐di‐GMP. All ITC‐measurements were carried out in 50 mM Tris‐HCl pH 7.5, 200 mM sodium chloride, 1 mM β‐mercaptoethanol at 20 °C using a MicroCal iTC200 instrument (Malvern Pananalytical, UK). In total 40 μl c‐di‐GMP was titrated to 270 μl protein after an initial delay of 120 s in 20 injections while stirring the sample with 750 rpm. The first injection of 0.2 μl c‐di‐GMP in 0.4 s was followed by 19 successive injections of 2 μl in 4 s separated by a 180 s delay using a reference power of 11 μcal^−1^. The feedback mode was set to high. Protein and c‐di‐GMP concentrations ranged from 30 μM to 100 μM and 300 μM to 1000 μM, respectively, and were adjusted to the respective binding affinities of the corresponding constructs to optimize measurement precision. Processing of the thermograms was carried out with Origin 7.0 (OriginLab) using a one‐site binding model. An overview of all ITC measurements is given in Supplementary Table 1.

### Circular Dichroism Spectroscopy

To investigate structural integrity as well as stability of the PilF_1–65_ and PilF_9–65_ constructs CD spectra and melting profiles were recorded on a Jasco‐815 CD spectrometer (Jasco, Gross‐Umstadt, Germany) and processed with OriginPro 2020 (OriginLab). All samples were prepared in 50 mM Tris‐HCl at pH 7.5, 20 mM NaCl and 1 mM β‐mercaptoethanol with a concentration of 40 μM and the experiments were conducted in 1 mm quartz cuvettes. The CD spectra were recorded as quintuples at 20 °C in a spectral range from 190 nm to 320 nm with a scanning speed of 200 nm min^−1^ in 0.5 nm scanning intervals with a bandwidth of 1 nm. Baseline correction and averaging of the five measurements were automatically performed. CD melting profiles were recorded in a temperature range from 20 °C to 100 °C with a temperature ramp of 1 °C min^−1^ at a constant wavelength (220 nm). Melting points (Tm) were calculated by fitting the recorded data points using the Boltzmann equation (Equation 1):

$$y=b+\frac{a-b}{1+\exp\left(\frac{T-Tm}{dx}\right)}$$

where a is the minimal and b the maximum value of the CD ellipticity, T is the Temperature, Tm is the melting temperature and dx is the slope.^[72]^

### NMR Spectroscopy

For NMR experiments, all PilF_1–154_, PilF_1–65_ and PilF_9–65_ samples were prepared in a buffer containing 50 mM BisTris‐HCl pH 5.8, 200 mM NaCl, 1 mM β‐mercaptoethanol with the addition of 5 % (v/v) D_2_O. 200 μM 2,2‐dimethyl‐2‐silapentane‐5‐sulfonic acid (DSS) was used to directly reference the ^1^H chemical shifts internally. With the appropriate conversion factors, the heteronuclear ^13^C, ^15^N and ^31^P chemical shifts were referenced indirectly.^[73]^ All spectra were processed and referenced with Bruker TopSpin™ versions 3.5 and 4.0.7. Chemical shifts were assigned in CARA.^[74]^ NMR‐spectra were acquired on Bruker AVANCE 600, 700, 800, 900 and 950 MHz spectrometers equipped with cryogenic triple resonance probes at 45 °C for PilF_1–154_ constructs and at 20 °C for the PilF_1–65_ and the PilF_9–65_ constructs.

For the backbone assignment of PilF_1–154_ a set of 3D‐BEST‐TROSY‐HNCO, ‐HNCACB and a 3D‐HN(CA)CO spectra were recorded with a 440 μM sample in H_2_O using standard pulse sequences,[^75^, ^76^] Side chain assignments were derived from 3D‐HBHA(CBACACO)NH, −CBCA(CO)NH, −HC(C)H‐TOCSY and −(H)CCH‐TOCSY spectra with a 340 μM sample in H_2_O supported by 3D−H(CCO)NH and 3D‐(H)C(CCO)NH experiments with the 440 μM sample in H_2_O.^[77]^ Stereospecific assignment of the L and V methyl groups (δ1, δ2 and γ1, γ2, respectively) was achieved by recording a standard ^13^C‐HSQC spectrum optimized for the aliphatic region with the stereo‐specifically labeled sample (608 μM) in H_2_O.^[60]^ The resonance assignments were deposited in the biological magnetic resonance bank (BMRB ID: 34929).

Protein backbone chemical shifts of PilF_1–65_ H33Q S39R A41G I55L I59L R62Q F63Y in the apo state and PilF_9–65_ H33Q S39R A41G I55L I59L R62Q F63Y bound to c‐di‐GMP were assigned with standard 3D‐BEST‐TROSY‐HNCO and ‐HNCACB spectra using samples containing either 500 μM PilF_1–65_ H33Q S39R A41G I55L I59L R62Q F63Y or 100 μM PilF_9–65_ H33Q S39R A41G I55L I59L R62Q F63Y and 5 equivalents (500 μM) of c‐di‐GMP in H_2_O,[^75^, ^76^] respectively. For these spectra non‐uniform sampling (NUS) with 25 % sparse sampling was applied.

Standard Bruker pulse sequences were used to analyze the fast time scale dynamics of PilF_1–154_ by recording {^1^H},^15^N‐heteronuclear Overhauser effect (hetNOE) experiments using 500 μM ^15^N‐labeled PilF_1–154_. The experiments were conducted twice in succession with and without proton saturation during the recovery delay.^[78]^ Peak volumes were acquired by integration with TopSpin™ 4.0.7 and hetNOE intensities (ΔI) were calculated using the following equation (Equation 3):

$$\Delta I=\frac{I_{x}}{I_{0}}$$

where I_X_ is the peak intensity with proton saturation and I_0_ without proton saturation.

^15^N spin relaxation experiments (longitudinal (R_1_) and transversal (R_2_) relaxation) were performed on a uniformly ^15^N labeled sample (340 μM) at 600 MHz in an interleaved fashion.^[78]^ To obtain R_1_ relaxation rates the experiments were run with delays of 20, 60, 100, 200, 400, 600, 800, 1200, 250, 600, 150 and 1600 ms in this order. R_2_ relaxation rates were acquired with delays of 17, 34, 68, 136, 170, 204, 272, 51, 272, 102, 34 and 85 ms in this order. Peak volumes were taken in TopSpin™ 4.0.7, plotted against the decay times and fitted using the following function (Equation 4):

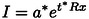




In Equation (3) a is the peak volume at 0 ms decay time, t is the relaxation decay time and R_x_ is either R_1_ or R_2_.

NMR titration experiments were carried out as ligand binding assays with uniformly ^15^N labeled protein samples from 50 μM to 100 μM and unlabeled c‐di‐GMP. A series of ^15^N‐HSQC, ^1^H,^15^N‐SOFAST‐HMQC or ^15^N‐BEST‐TROSY‐HSQC spectra were recorded with increasing c‐di‐GMP concentrations (up to 1.4 mM) and chemical shift changes were evaluated with TopSpin™ 4.0.7.

### CLEANEX‐PM

To analyze water‐amide exchange on a millisecond time‐scale a series of phase‐modulated CLEAN chemical exchange (CLEANEX‐PM) experiments (mixing times 5, 10, 15, 20 and 150 ms) in combination with ^1^H,^15^ N‐FHSQC spectra for detection were conducted at 45 °C on a Bruker AVANCE 800 MHz spectrometer using uniformly ^15^N‐labeled samples of PilF_1–154_ (1 mM) and PilF_159–302_ (1 mM).^[79]^

### Direct Detection of Intermolecular Hydrogen Bonds

To investigate intermolecular hydrogen bonding interactions a set of two optimized NMR‐experiments was carried out. Initially, a 2D‐^1^H‐^31^P SOFAST‐HMQC spectrum optimized for magnetization transfer across a hydrogen bond starting from an amide proton (H^N^) as the donor to the acceptor phosphate group of the respective GMP moiety was used.[^64^, ^65^] For this experiment a sample of 350 μM unlabeled PilF_9–65_ F63Y H33Q S39R A41G I55L I59L R62Q with 700 μM c‐di‐GMP was used. In addition, a 2D‐BEST‐HNN‐COSY experiment optimized for RNA was conducted to investigate possible ^H^N to N7guanine hydrogen bonds with a sample containing 445 μM ^15^N‐PilF_9–65_ F63Y H33Q S39R A41G I55L I59L R62Q with 890 μM of ^13^C ^15^N labeled c‐di‐GMP.[^80^, ^81^] To identify the hydrogen bond acceptor an lr‐^15^N‐HSQC optimized for RNA with a transfer delay of 14.2 ms^[82]^ was used to transfer magnetization from H8 to N7 and N9 in the guanine base moieties.

### Structure Calculation

NMR structures of PilF_1–154_ were calculated with distance restraints based on the nuclear Overhauser effect (NOE) as well as torsion angle restraints extracted from backbone HN, N, Cα, Cβ and CO chemical shifts using TALOS‐N.^[59]^ The backbone chemical shifts were also used for the identification of helix capping motifs using the web‐based tool MISC.^[61]^ Distance restraints were extracted from ^15^N‐NOESY‐HSQC and ^13^C‐NOESY‐HSQC experiments optimized for the aliphatic and aromatic regions using a 340 μM uniformly ^13^C,^15^N labeled sample in H_2_O with mixing times of 120 ms. Additional distance restraints were obtained from a ^13^C‐NOESY‐HSQC experiment with the ^13^C offset optimized for leucine and valine methyl groups (δ1, δ2 and γ1, γ2 respectively) with a 560 μM uniformly ^15^N and L/V methyl group ^13^C labeled sample in H_2_O with a mixing time of 150 ms. All NOE peaks were picked manually and evaluated in CcpNmr‐Analysis.^[83]^ NOE assignment and distance restraint extraction based on the 3D NOESY spectra as well as structure calculation was performed in an automated fashion by CYANA.^[84]^ The resulting peak list was reviewed manually and corrected in case of artifacts. A small number of ambiguous NOE assignments were evaluated and corrected manually in CcpNmr‐Analysis^[83]^ and CYANA.^[84]^ For all NOESY spectra except the ^13^C‐NOESY of the aromatic region (53 %) an assignment of >80 % of the observable NOE signals was achieved. In the final structure calculation by CYANA the 20 structures with the lowest target function, out of 100 structures total calculated in in each run, were used for energy refinement with OPALp.^[85]^ The atomic coordinates of the final structure ensemble of the 20 lowest energy structures were deposited in the protein data bank (PDB ID: 9G1W). All structure figures were exported from PyMOL.

## Supporting Information Summary

Supplementary data (Supplementary Table 1 and Supplementary Figures 1–17) are available in the Supporting Information.

## Conflict of Interests

The authors declare no conflict of interest.

1

## Supporting information

As a service to our authors and readers, this journal provides supporting information supplied by the authors. Such materials are peer reviewed and may be re‐organized for online delivery, but are not copy‐edited or typeset. Technical support issues arising from supporting information (other than missing files) should be addressed to the authors.

Supporting Information

## Data Availability

The structural data reported in this paper are publicly available from the protein databank under accession code 9G1W. The NMR resonance assignments were deposited in the biological magnetic resonance data bank with the BMRB ID: 34929.
