# Supplementary material for: NMR Solution Structure of the N‐Terminal GSPII Domain from the Thermus Thermophilus Traffic ATPase PilF and Reconstruction of its c‐di‐GMP Binding Capability
Source: Chembiochem. 2025 Mar 12;26(7):e202400959. doi: 10.1002/cbic.202400959 (PMC12002112; doi:10.1002/cbic.202400959)
Supplement: Supplementary file 1 — Supporting Information [file CBIC-26-e202400959-s001.pdf]

ChemBioChem

Supporting Information

NMR Solution Structure of the N-Terminal GSPII Domain from the *Thermus Thermophilus* Traffic ATPase PilF and Reconstruction of its c-di-GMP Binding Capability

Konstantin Neißner, Carolin Frohnapfel, Heiko Keller, Elke Duchardt-Ferner, Vanessa Schneider, Zeinab Kamjou, Beate Averhoff, and Jens Wöhnert*

Supplementary Material

NMR Solution Structure of the N-terminal GSPII Domain from the *Thermus thermophilus* Traffic ATPase PilF and Reconstruction of its c-di-GMP Binding Capability

Konstantin Neißner[b,c], Carolin Frohnapfel[b,c,d], Heiko Keller[b,c], Elke Duchardt-Ferner[b,c], Vanessa Schneider[b,c], Zeinab Kamjou[b,c], Beate Averhoff[b,e], Jens Wöhnert*[a,b,c]

---

[a] Prof. Dr. J, Wöhnert
Institute for Molecular Biosciences
Goethe-University Frankfurt/M.
Max-von-Laue-Str. 9, 60438 Frankfurt, Germany
woehnert@bio.uni-frankfurt.de

[b] Prof. Dr. J, Wöhnert, Prof. Dr. B. Averhoff, K. Neißner, Dr. C. Frohnapfel, Dr. H. Keller, Dr. E. Duchardt-Ferner, V. Schneider, Z. Kamjou
Institute for Molecular Biosciences
Goethe-University Frankfurt/M.
Max-von-Laue-Str. 9, 60438 Frankfurt, Germany

[c] Prof. Dr. J, Wöhnert, K. Neißner, Dr. C. Frohnapfel, Dr. H. Keller, Dr. E. Duchardt-Ferner, V. Schneider, Z. Kamjou
Center for Biomolecular Magnetic Resonance (BMRZ)
Goethe-University Frankfurt/M.
Max-von-Laue-Str. 9, 60438 Frankfurt, Germany

[d] Dr. C. Frohnapfel
Bruker BioSpin GmbH & Co. KG
Rudolf-Plank-Str. 23, 76275 Ettlingen, Germany

[e] Prof. Dr. B. Averhoff
Molecular Microbiology and Bioenergetics
Goethe-University Frankfurt/M.
Max-von-Laue-Str. 9, 60438 Frankfurt, Germany

Supporting data

Supplementary Table 1: ITC data for PilF constructs. Non-binding is indicated by /.

Construct	Ligand	Temperature [°C]	N	K_D	ΔH [cal/mol]	ΔS [cal/mol/deg]
**PilF$_{1-889}$**	c-di-GMP	20	1 = 0.72 ± 0.1<br>2 = 0.59 ± 0.03	1 = 324 ± 2 nM<br>2 = 10 ± 5 nM	1 = -11763 ± 780<br>2 = -15683 ± 970	1 = -10.4 ± 2.7<br>2 = -16.5 ± 2.2
**PilF$_{1-154}$**	c-di-GMP	20	/	/	/	/
**PilF$_{1-65}$ F63Y**	c-di-GMP	20	/	/	/	/
**PilF$_{1-65}$ F63Y H33Q S39R A41G I55L I59L R62Q**	c-di-GMP	20	0.6 ± 0.2	13 ± 2 µM	-1342 ± 513	17.1 ± 2.3
**PilF$_{9-65}$ F63Y H33Q S39R A41G I55L I59L R62Q**	c-di-GMP	20	1.2 ± 0.1	695 ± 42 nM	-12953 ± 441	-17.9 ± 1.4
**PilF$_{9-65}$ F63Y H33Q S39R A41G R62Q**	c-di-GMP	20	0.8 ± 0.1	1.8 ± 0.3 µM	-10127 ± 499	-6.4 ± 1.7
**PilF$_{9-65}$ F63Y H33Q S39R R62Q**	c-di-GMP	20	0.7 ± 0.1	19 ± 6 µM	-9574 ± 243	-3.6 ± 0.7
**PilF$_{9-65}$ F63Y H33Q R62Q**	c-di-GMP	20	/	/	/	/

Supplementary Figure 1

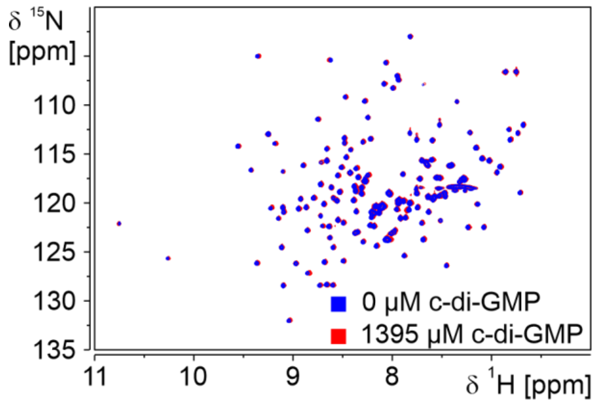

Supplementary Fig. 1: Overlay of the ^{1}H,^{15}N-SOFAST-HMQC spectra of PilF$_{1\text{-}154}$ H33Q S39R A41G I55L I59L R62Q (100 µM) in the absence (blue) and the presence of 15 equivalents (red) of c-di-GMP at 800 MHz.

Supplementary Figure 2

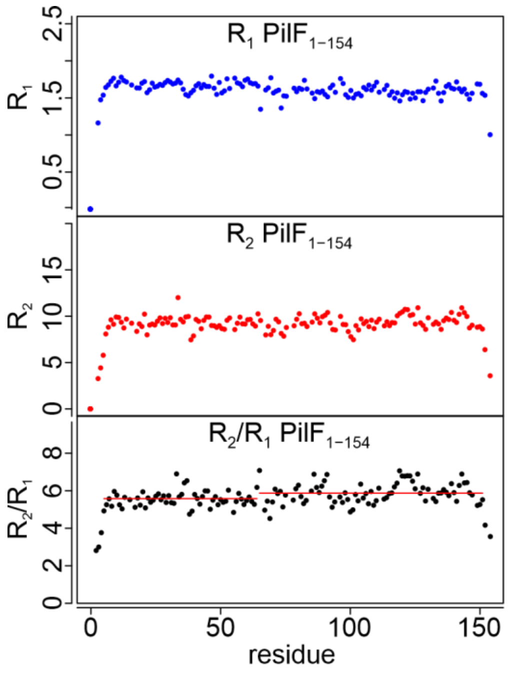

Supplementary Fig. 2: Longitudinal (R_1) and transversal (R_2) relaxation rates of $PilF_{1\text{-}154}$ plotted against the sequence. The red bars in the bottom diagram represent the median of the R_2/R_1 ratio of the N- and C-terminal subdomains.

Supplementary Figure 3

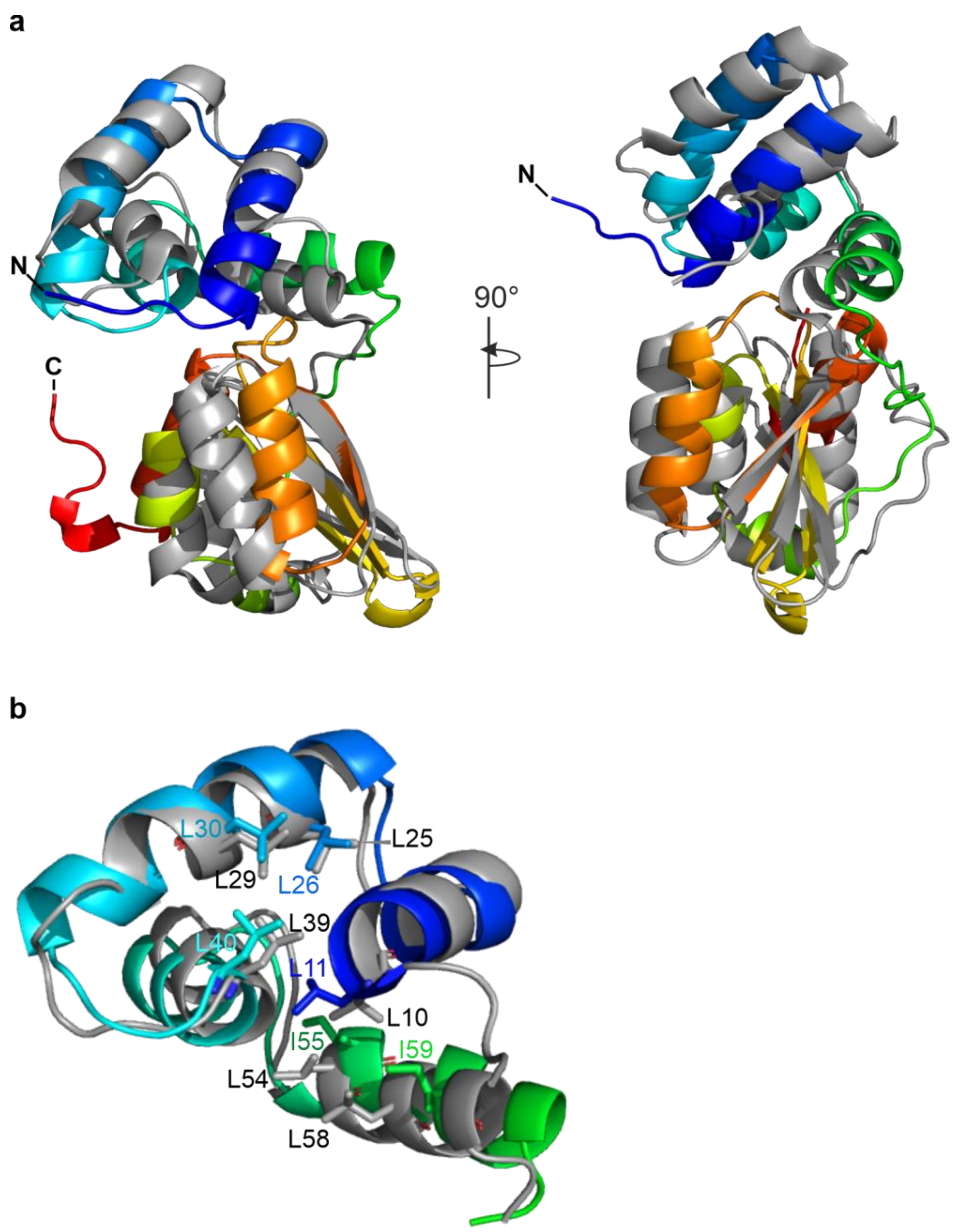

Supplementary Fig. 3: (a) Global alignment of $PilF_{1\text{-}154}$ (rainbow) and MshEN (gray) with an overall RMSD of 3.9 Å in two orientations. **(b)** Alignment of the N-terminal subdomains of $PilF_{1\text{-}154}$ (rainbow) and MshEN (gray) (RMSD 1.7 Å). The leucine residues that form the two triangular hydrophobic cores that are essential for c-di-GMP binding in MshEN are presented as sticks. The leucine and isoleucine residues at the corresponding positions in $PilF_{1\text{-}154}$ are also presented as sticks. Residue positions are given according to the MshEN (black) or $PilF_{1\text{-}154}$ (rainbow) sequence.

Supplementary Figure 4

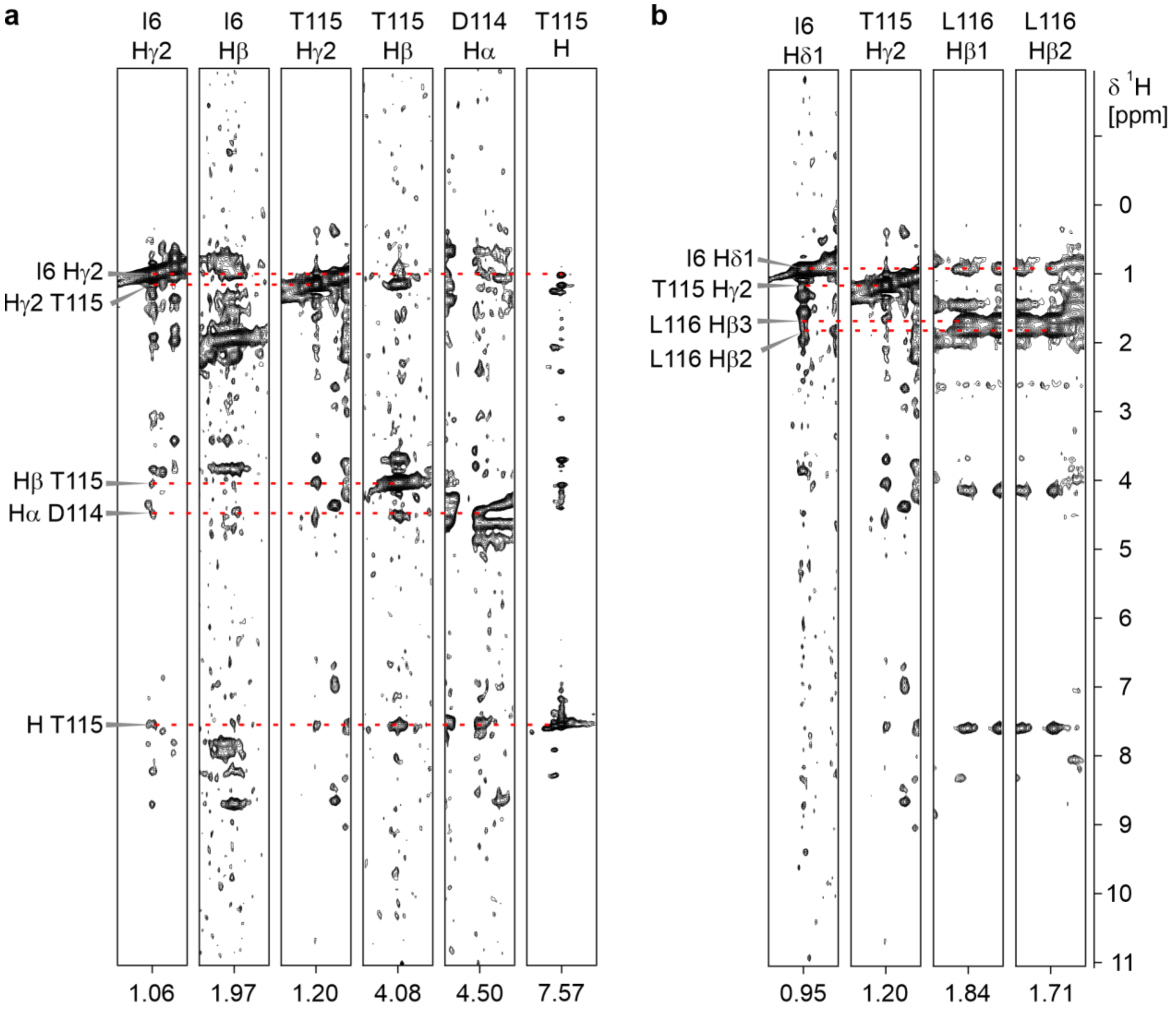

Supplementary Fig. 4: (a) NOE cross peaks between residues I6 from helix α1 and D114 as well as T115 from helix α7 showing the spatial proximity of these residues. **(b)** Additional NOEs between I6 (helix α1) and T115 as well as L116 (helix α7) supporting the spatial proximity of these residues and the corresponding helices. The NOE slices were extracted from ^{15}N-NOESY-HSQC and ^{13}C-NOESY-HSQC experiments that were recorded with uniformly ^{13}C,^{15}N labeled PilF$_{1\text{-}154}$ in H_2O at 45°C (600 MHz).

Supplementary Figure 5

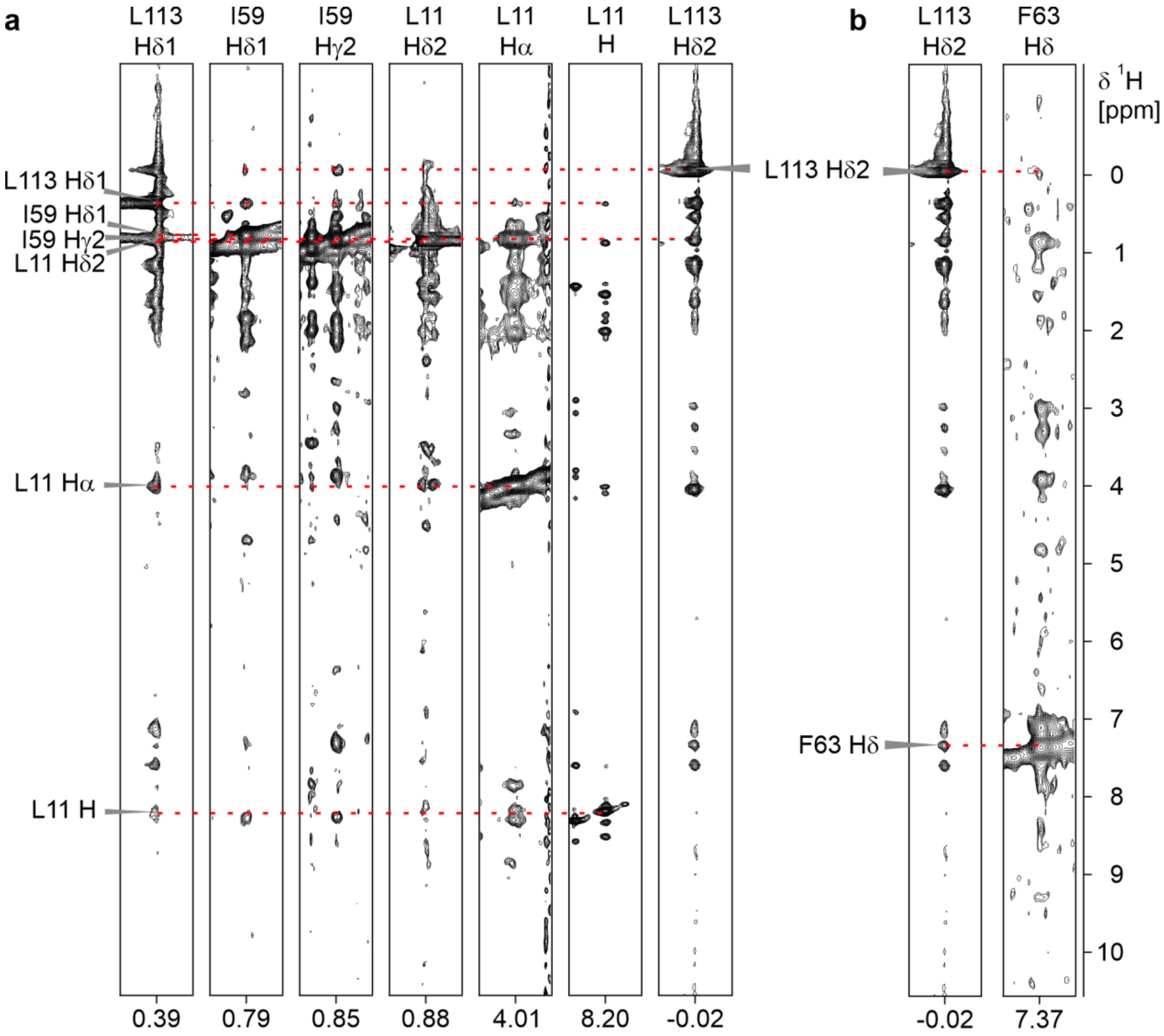

Supplementary Fig. 5: (a) NOE cross peaks between residues L113, I59 and L11 supporting the placement of L113 of the C-terminal subdomain near the core of the N-terminal subdomain. **(b)** Reciprocal NOEs between L113 Hδ2 and F63 Hδ in the N-terminal subdomain. The NOE slices (except L113) were extracted from ^{15}N-NOESY-HSQC and ^{13}C-NOESY-HSQC experiments that were recorded with uniformly ^{13}C,^{15}N labeled $PilF_{1\text{-}154}$ in H_2O at 45°C. The NOE slices of L113 were extracted from a ^{13}C-NOESY-HSQC experiment with the ^{13}C offset optimized for leucine and valine methyl groups (δ^1, δ^2 and γ^1, γ^2 respectively) with a uniformly ^{15}N and L/V methyl group ^{13}C labeled sample in H_2O at 45°C (600 MHz).

Supplementary Figure 6

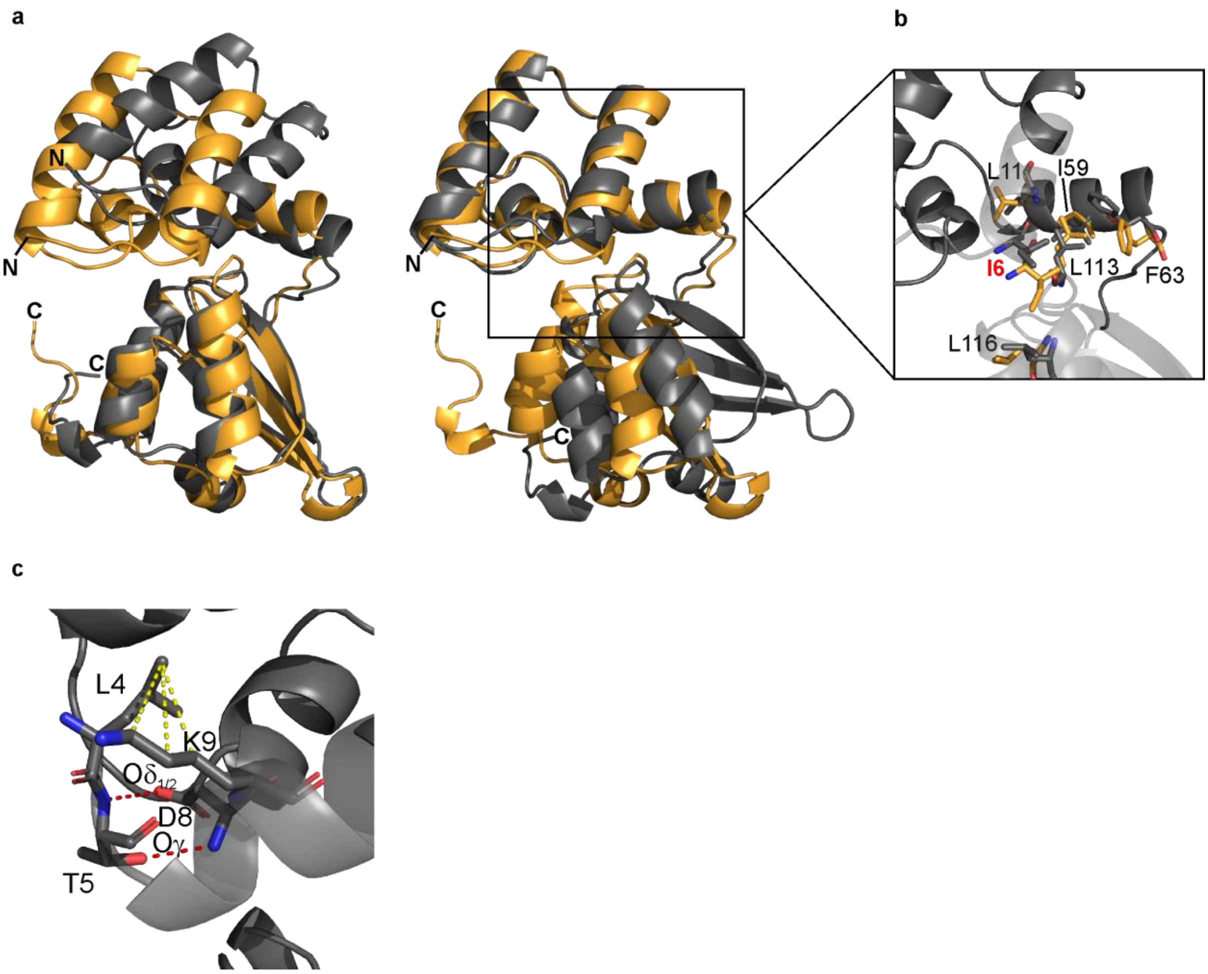

Supplementary Fig. 6: (a) Alignment of the C-terminal (left) and N-terminal subdomains (right) of the PilF$_{1\text{-}154}$ NMR-structure (orange) with the AlphaFold3 model (dark gray). The subdomains align with backbone RMSD values of 1.2 Å (C-terminal subdomains) and 0.9 Å (N-terminal subdomains). **(b)** Comparison of the subdomain contacts in the NMR-structure (orange) and the AlphaFold3 model (dark gray). The involved residues of the AlphaFold3 model are depicted in red to enhance visibility. **(c)** Close-up of the Ncap motif in the AlphaFold 3 model. Red and yellow dashed lines indicate hydrogen bonding and hydrophobic interactions, respectively.

Supplementary Figure 7

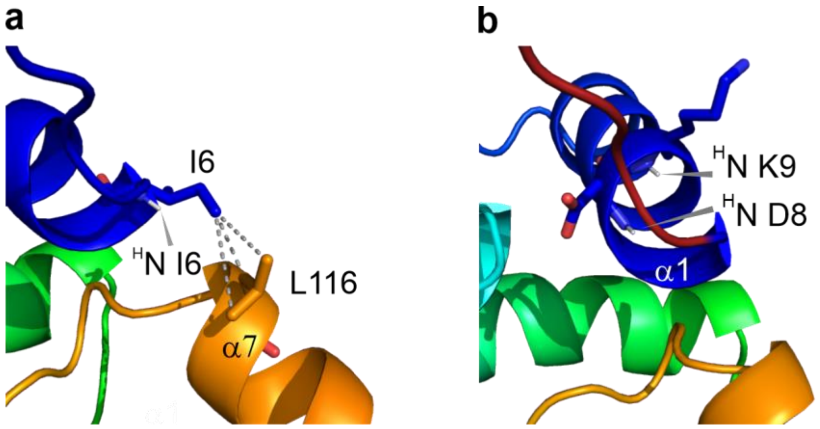

Supplementary Fig. 7: (a) Hydrophobic interaction between I6 of helix α1 and L116 (sticks) of helix α7 resulting in a close contact of the two helices. **(b)** Close up of the N-terminal tip of helix α1 with the loop (red) preceding this helix shielding the ^{H}N groups of D8 and K9 from the solvent.

Supplementary Figure 8

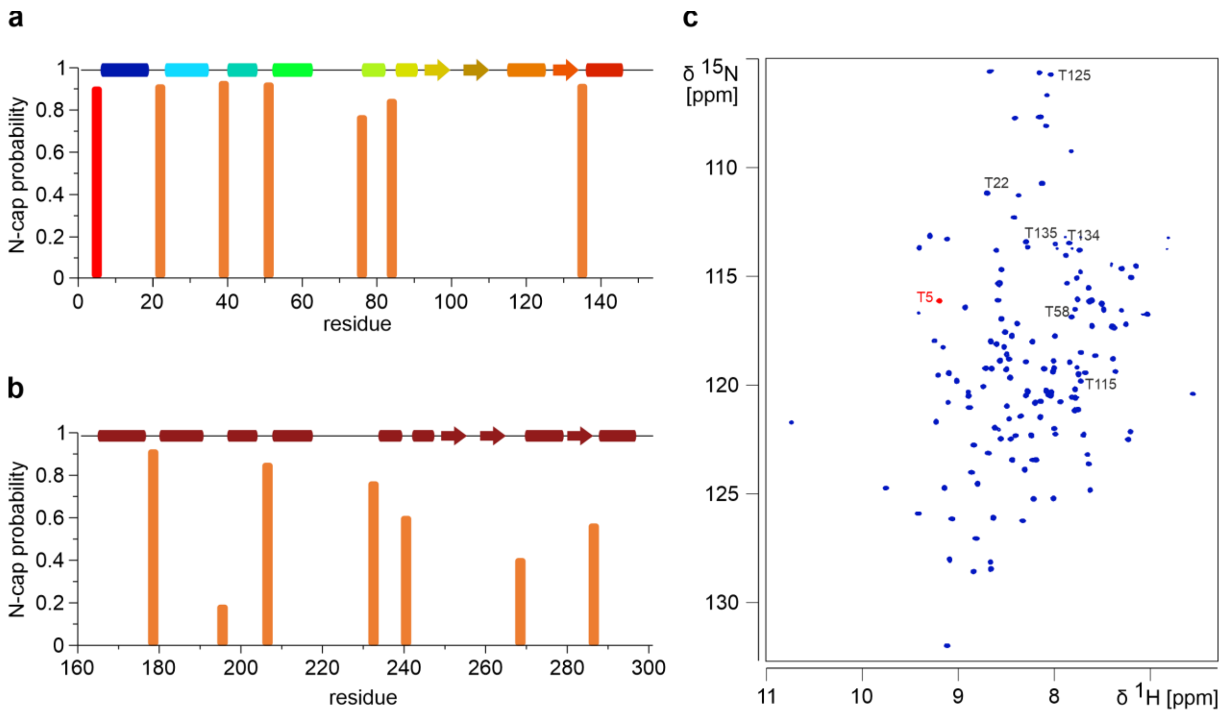

Supplementary Fig. 8: (a) Chemical shift based Ncap probability calculation for PilF$_{1-154}$ plotted against the full-length sequence. The first Ncap probability bar is colored red to indicate the presence of the Ncap in PilF$_{1-154}$ as opposed to PilF$_{159-302}$. Secondary structure elements are presented as cartoon representation at the top. α-helices are depicted as cylinders and β-sheets as arrows. The colors correspond to the rainbow depiction of the 3D-structure shown in Fig. 2. **(b)** Chemical shift based Ncap probability calculation for PilF$_{159-302}$ plotted against the sequence with a cartoon scheme of the secondary structure elements shown at the top. **(c)** ^{1}H,^{15}N-BEST-TROSY-HSQC spectrum of ^{13}C,^{15}N PilF$_{1-154}$ (438 µM) at 45°C (600 MHz). The position of the amide resonance of T5 in the spectrum is highlighted in red. For comparison, the positions of the other threonine amide group resonances are labeled in black.

Supplementary Figure 9

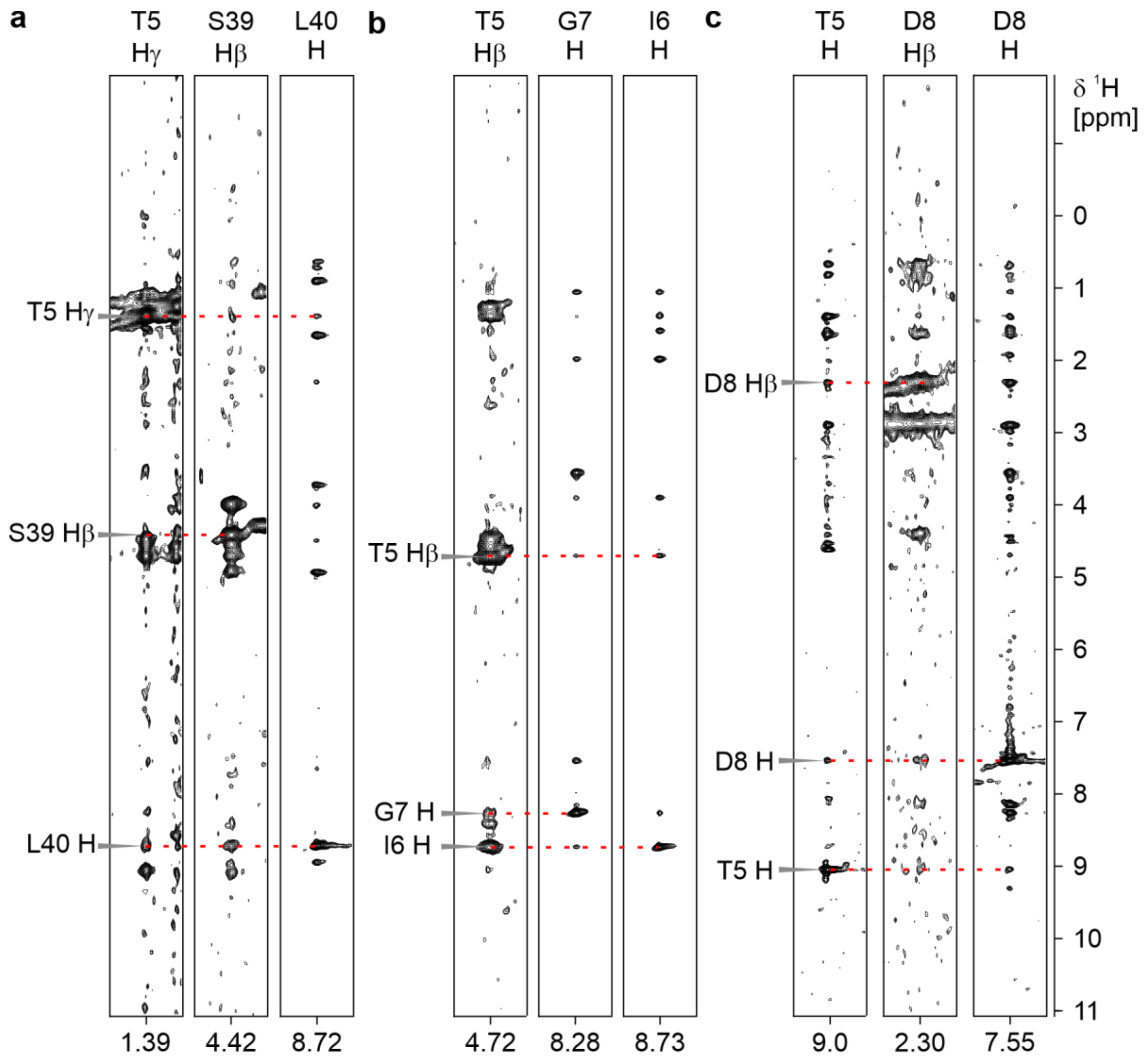

Supplementary Fig. 9: NOE cross peak patterns for T5, I6, G7, S39 and L40 showing the spatial proximity of T5 Hγ to S39 and L40 of helix α4 (a) and T5 Hβ to I6 and G7 (b). (c) NOE-pattern between T5 and D8 showing the close spatial proximity of these two residues. The NOE slices were extracted from ^{15}N-NOESY-HSQC and ^{13}C-NOESY-HSQC experiments that were recorded with uniformly ^{13}C,^{15}N labeled PilF$_{1\text{-}154}$ in H_2O at 45°C (600 MHz).

Supplementary Figure 10

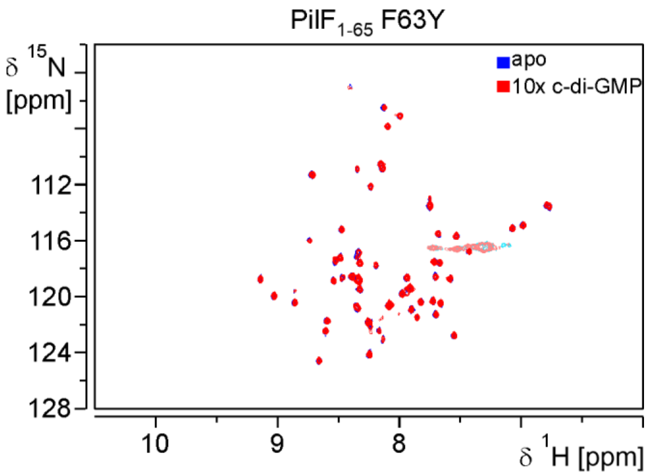

Supplementary Fig. 10: ^{1}H,^{15}N-SOFAST-HMQC spectra of PilF$_{1\text{-}65}$ F63Y (48 µM) in the absence (blue) and the presence of 10 equivalents (480 µM) of c-di-GMP (red) at 20°C (800 MHz).

Supplementary Figure 11

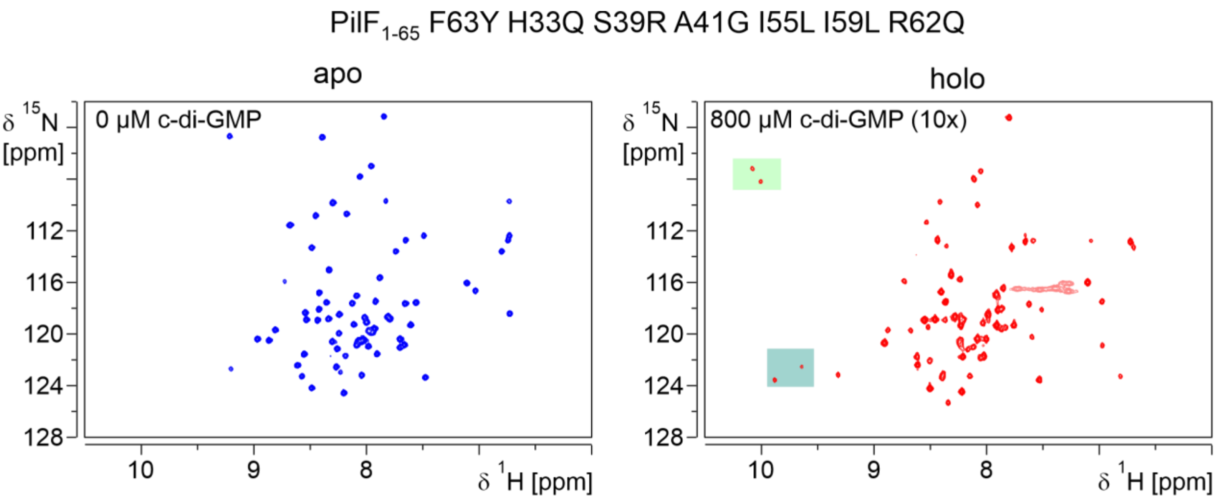

Supplementary Fig. 11: c-di-GMP binding of PilF$_{1-65}$ F63Y H33Q S39R A41G I55L I59L R62Q. ^{1}H,^{15}N-HSQC spectra of the apo- (left, blue) and the holo-state (right, red) of PilF$_{1-65}$ F63Y H33Q S39R A41G I55L I59L R62Q at 20°C (800 MHz). The colored regions indicate L11, G12, L40 and G41 resonances, respectively. For the apo state an 83 µM sample and for the holo state a 90 µM sample with 800 µM c-di-GMP (10x excess) was used.

Supplementary Figure 12

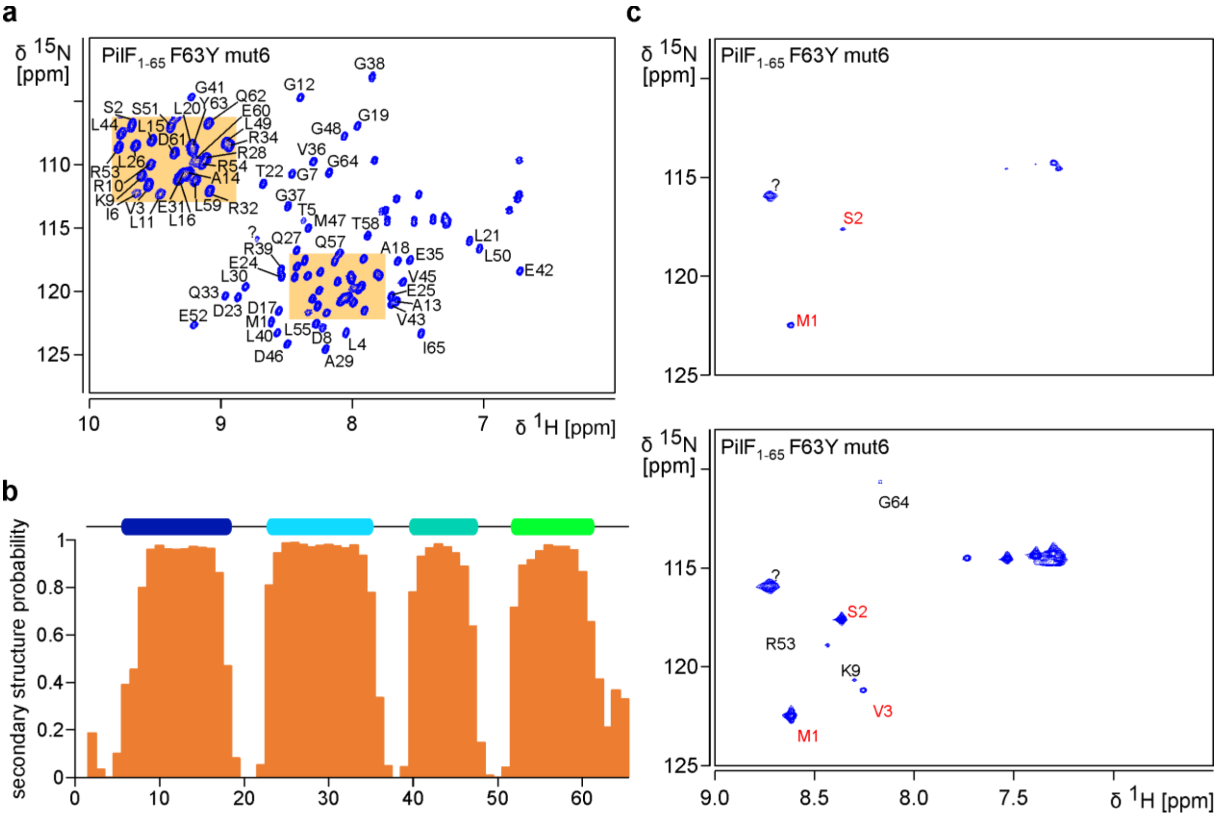

Figure 12: (a) Backbone amide resonance assignment of PilF$_{1-65}$ F63Y H33Q S39R A41G I55L I59L R62Q in the absence of c-di-GMP. A ^{1}H,^{15}N-HSQC spectrum of 500 µM ^{13}C,^{15}N PilF$_{1-65}$ F63Y H33Q S39R A41G I55L I59L R62Q at 20°C (600 MHz) with all amide group resonances assigned to their respective residue is shown. Assignments for resonances located in the orange square are show as an inset in the upper left corner of the spectrum. **(b))** Chemical shift based secondary structure of PilF$_{1-65}$ F63Y H33Q S39R A41G I55L I59L R62Q in the absence of c-di-GMP calculated by TALOS-N [76]. **(c)** CLEANEX-PM spectra of PilF$_{1-65}$ F63Y H33Q S39R A41G I55L I59L R62Q in with mixing times of 5 ms (top) and 150 ms (bottom) at 600 MHz. The N-terminal residues of PilF$_{1-65}$ F63Y H33Q S39R A41G I55L I59L R62Q are highlighted in red in both panels

Supplementary Figure 13

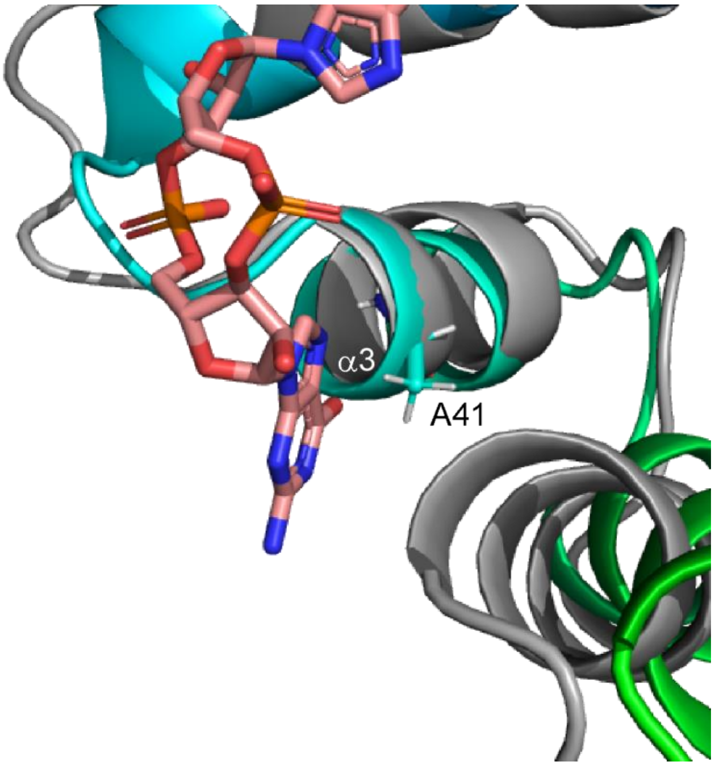

Supplementary Fig. 13: Close up of c-di-GMP bound to MshEN (gray) with the N-terminal subdomain of PilF$_{1-154}$ (rainbow) aligned. The A41 side chain of PilF$_{1-154}$ which potentially occupies part of the putative c-di-GMP binding pocket is shown in a stick presentation.

Supplementary Figure 14

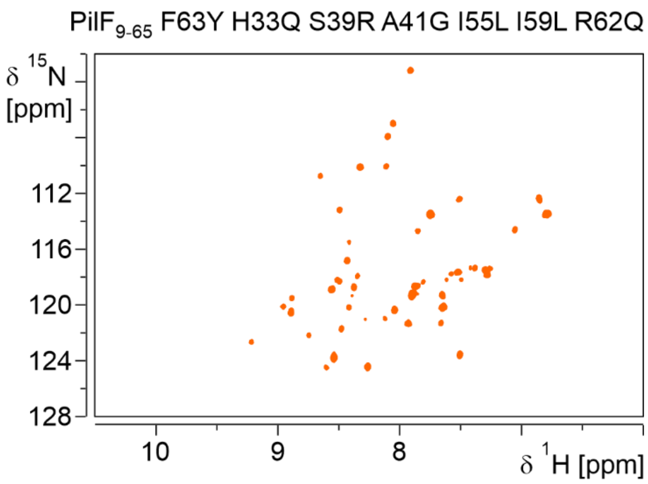

Supplementary Fig. 14: ^{1}H,^{15}N-HSQC spectrum of PilF$_{9\text{-}65}$ F63Y H33Q S39R A41G I55L I59L R62Q (72 µM) in the absence of c-di-GMP at 20°C (600 MHz).

Supplementary Figure 15

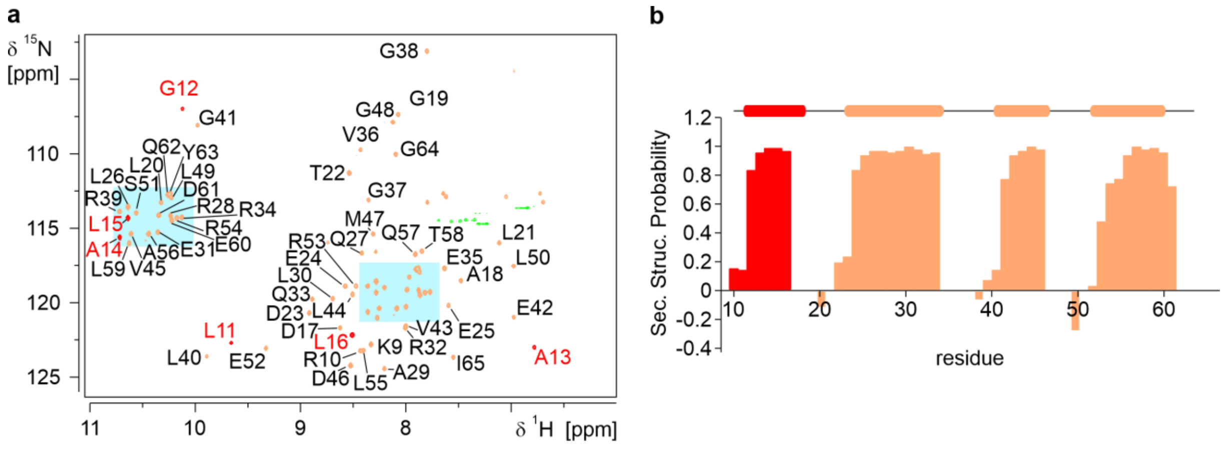

Supplementary Fig. 15: (a) Backbone amide resonance assignment of PilF$_{9\text{-}65}$ F63Y H33Q S39R A41G I55L I59L R62Q bound to c-di-GMP. A ^{1}H,^{15}N-SOFAST-HMQC spectrum of 100 µM ^{13}C,^{15}N PilF$_{9\text{-}65}$ F63Y H33Q S39R A41G I55L I59L R62Q with 500 µM c-di-GMP at 20°C (600 MHz) with all amide group resonances assigned to the respective residue is shown. The resonances of helix α1 are highlighted in red. **(b)** Chemical shift based secondary structure of PilF$_{9\text{-}65}$ F63Y H33Q S39R A41G I55L I59L R62Q bound to c-di-GMP calculated by TALOS-N [76]. Helix α1 is highlighted in red and α-helices are presented as cartoon cylinder representation.

Supplementary Figure 16

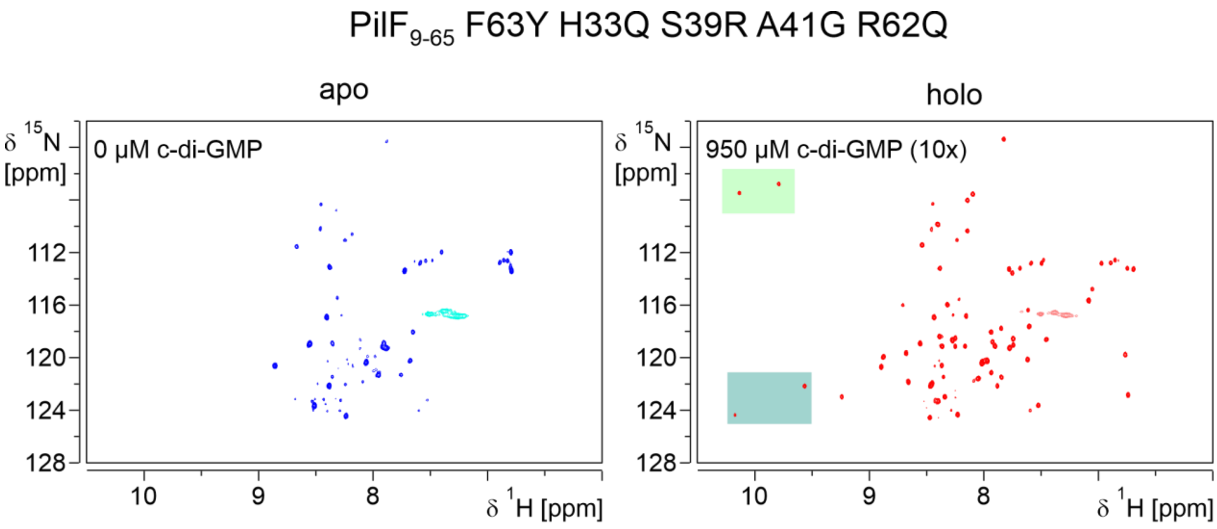

Supplementary Fig. 16: ^{1}H,^{15}N-SOFAST-HMQC spectra of the apo- (left, blue) and the holo-state (right, red) of PilF$_{9\text{-}65}$ F63Y H33Q S39R A41G R62Q at 20°C (800 MHz). The colored regions indicate the L11, G12, L40 and G41 resonances. For the apo state a 100 µM sample and for the holo state a 95 µM sample with 950 µM c-di-GMP (10x excess) was used.

Supplementary Figure 17

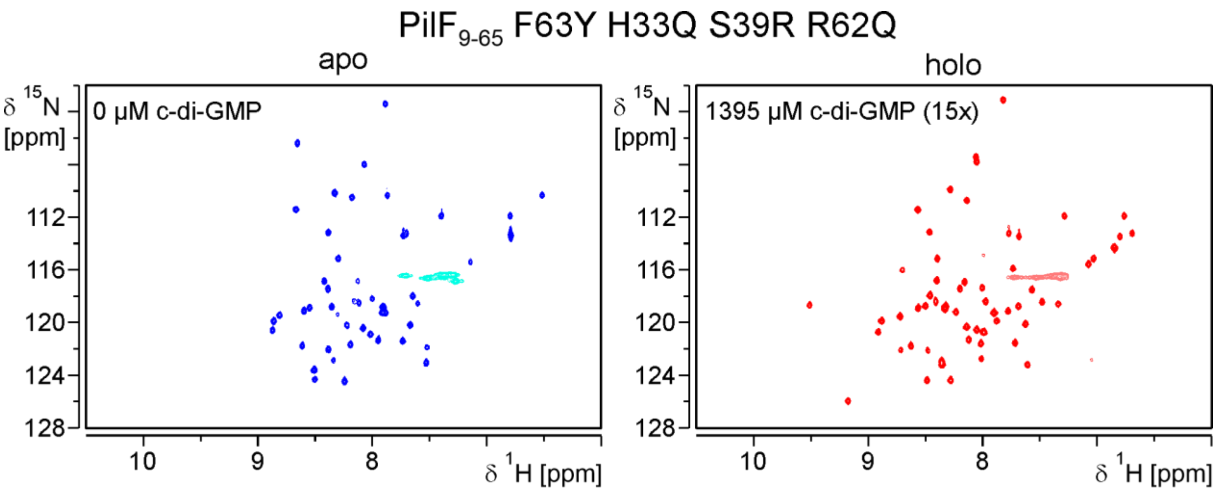

Supplementary Fig. 17: ^{1}H, ^{15}N-SOFAST-HMQC spectra of the apo- (left, blue) and the holo-state (right, red) of ^{15}N PilF9$_{-65}$ F63Y H33Q S39R R62Q at 20°C (800 MHz). For the apo state a 96 µM sample and for the holo state a 93 µM sample with 1395 µM c-di-GMP (15fold excess) was used.
